# MERS-CoV 4b protein interferes with the NF-κB-dependent innate immune response during infection

**DOI:** 10.1371/journal.ppat.1006838

**Published:** 2018-01-25

**Authors:** Javier Canton, Anthony R. Fehr, Raúl Fernandez-Delgado, Francisco J. Gutierrez-Alvarez, Maria T. Sanchez-Aparicio, Adolfo García-Sastre, Stanley Perlman, Luis Enjuanes, Isabel Sola

**Affiliations:** 1 Department of Molecular and Cell Biology, Centro Nacional de Biotecnología (CNB-CSIC), Madrid, Spain; 2 Department of Microbiology and Immunology, University of Iowa Carver College of Medicine, Iowa City, IA, United States of America; 3 Department of Microbiology, Icahn School of Medicine at Mount Sinai, New York, NY, United States of America; 4 Global Health and Emerging Pathogens Institute. Icahn School of Medicine at Mount Sinai, New York, NY, United States of America; 5 Department of Medicine, Division of Infectious Diseases, Icahn School of Medicine at Mount Sinai, New York, NY, United States of America; Georgia State University, UNITED STATES

## Abstract

Middle East respiratory syndrome coronavirus (MERS-CoV) is a novel human coronavirus that emerged in 2012, causing severe pneumonia and acute respiratory distress syndrome (ARDS), with a case fatality rate of ~36%. When expressed in isolation, CoV accessory proteins have been shown to interfere with innate antiviral signaling pathways. However, there is limited information on the specific contribution of MERS-CoV accessory protein 4b to the repression of the innate antiviral response in the context of infection. We found that MERS-CoV 4b was required to prevent a robust NF-κB dependent response during infection. In wild-type virus infected cells, 4b localized to the nucleus, while NF-κB was retained in the cytoplasm. In contrast, in the absence of 4b or in the presence of cytoplasmic 4b mutants lacking a nuclear localization signal (NLS), NF-κB was translocated to the nucleus leading to the expression of pro-inflammatory cytokines. This indicates that NF-κB repression required the nuclear import of 4b mediated by a specific NLS. Interestingly, we also found that both in isolation and during infection, 4b interacted with α-karyopherin proteins in an NLS-dependent manner. In particular, 4b had a strong preference for binding karyopherin-α4 (KPNA4), which is known to translocate the NF-κB protein complex into the nucleus. Binding of 4b to KPNA4 during infection inhibited its interaction with NF-κB-p65 subunit. Thereby we propose a model where 4b outcompetes NF-κB for KPNA4 binding and translocation into the nucleus as a mechanism of interference with the NF-κB-mediated innate immune response.

## Author summary

Middle East respiratory syndrome coronavirus (MERS-CoV) is a highly pathogenic human CoV that continues to cause lethal human infections, primarily in the Middle East. Virus accessory genes are potential contributors to pathology, possibly by interfering with the innate immune response. However, understanding their interactions with host proteins in the context of infection is rudimentary. Here, we provide evidence that the MERS-CoV accessory protein 4b functioned, at least in part, to prevent a robust NF-κB dependent response during infection. This effect depended on the nuclear localization of 4b, which was associated with the cytoplasmic retention of NF-κB. We show that 4b interacted with α-karyopherin proteins (importins) involved in the nuclear import of NF-κB, inhibiting the binding of α-karyopherin to NF-κB-p65 subunit. We propose that 4b contributes to the evasion of the innate immune response by binding α-karyopherin proteins, leading to the inhibition of NF-κB nuclear transport.

## Introduction

Middle East respiratory syndrome coronavirus (MERS-CoV) is a human coronavirus that emerged in the Middle East in 2012. Since then, 2100 confirmed cases and 730 deaths have been reported, with an estimated mortality rate of ~36% (http://www.who.int/csr/don/10-november-2017-mers-oman/en/). Currently, MERS-CoV remains a public health threat, as new infections continue to occur and no approved vaccines or antivirals are available. Furthermore, MERS-CoV is endemic in camels, making it likely that the virus will be an ongoing concern. Evidence from a number of studies has demonstrated that MERS-CoV strongly inhibits IFN-α/β production [1–3], although our knowledge on how the virus interferes with the host antiviral response is still limited. The contribution of viral proteins to the inhibition of the innate antiviral and pro-inflammatory responses has been mainly addressed in cells overexpressing individual viral proteins, not in the context of virus infection.

The nuclear factor-κB (NF-κB) family of proteins are critical regulators of both innate and adaptive immunity [4]. In naïve cells, NF-κB proteins are present in the cytoplasm in association with inhibitory proteins (IκBs). Cellular stimuli, including pathogens and stress, induce IκB phosphorylation, ubiquitination and degradation by the proteasome, releasing NF-κB proteins and allowing their nuclear translocation. In the nucleus, NF-κB proteins promote the transcription of pro-inflammatory cytokines and chemokines, stress-response proteins, and anti-apoptotic proteins. NF-κB activity is essential for lymphocyte survival and activation, and for initiating and propagating optimal immune responses. By contrast, the constitutive activation of the NF-κB pathway is often associated with inflammatory diseases, such as rheumatoid arthritis and asthma. Exacerbated NF-κB activity has been demonstrated to be important for the inflammatory immunopathology induced by respiratory viruses such as severe acute respiratory syndrome (SARS)-CoV [5, 6] and influenza A virus [7]. Here, we found that nuclear MERS-CoV 4b protein contributes to the inhibition of the host NF-κB-mediated pro-inflammatory response by preventing the nuclear translocation of NF-κB. In addition, we identified karyopherin-α4 (importin-α3), which is responsible for the nuclear import of NF-κB, as a 4b binding protein. We found that 4b binding to karyopherin-α4 inhibited its interaction with NF-κB-p65 subunit, thus preventing NF-κB nuclear translocation and, as a consequence, expression of NF-κB-dependent pro-inflammatory cytokines during infection. The ability of 4b to block NF-κB is likely to be relevant for MERS-CoV-induced *in vivo* pathology and virulence.

## Results

### Identification of MERS-CoV accessory genes that interfere with the innate immune response during infection

To address whether MERS-CoV accessory genes 3, 4a, 4b or 5 interfere with the innate immune response during infection, we infected Huh-7 cells with previously engineered MERS-CoV deletion mutants (Δ3, Δ4ab, Δ5) [8] and analyzed the expression of interferon β (IFN-β) and the pro-inflammatory cytokines IL-6, IL-8, and TNF-α. Viruses deleted in the accessory proteins, like the parental virus [3], were unable to induce IFN (S1 Fig) in contrast to the effective induction of IFN-β by poly(I:C) transfection, implying that additional proteins expressed by the virus act as IFN antagonists [9]. Interestingly, dual deletion of genes 4a and 4b led to a significant increase in the expression levels of IL-6, IL-8, and TNF-α as compared to the wild-type infection (S1 Fig). Since the transcription of these genes depends on NF-κB [4], these results suggested that one or both proteins repressed NF-κB activation during MERS-CoV infection.

### Contribution of MERS-CoV 4a and 4b proteins to the inhibition of NF-κB activity

**Fig 1:**
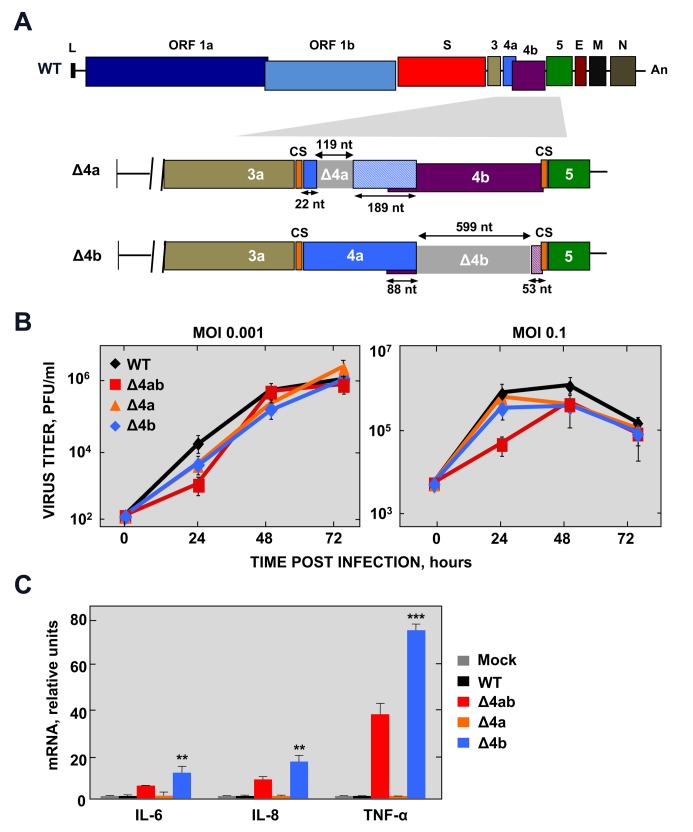
Generation of MERS-CoV-Δ4a and Δ4b deletion mutants. (A) Schematic representation of MERS-CoV WT genome. Essential viral genes (ORF1a, ORF1b, S, E, M, N) and accessory genes (3, 4a, 4b and 5) are indicated. L, leader sequence; A_n_, poly(A) tail; CS, conserved sequence included in transcription-regulating sequences. Genes 4a and 4b were deleted from the MERS-CoV infectious cDNA clone by PCR-directed mutagenesis as described in Materials and Methods. Deleted regions within 4a and 4b genes are illustrated as light grey boxes. The size of both deleted and remaining regions in 4a and 4b genes is indicated by arrows. The patterned boxes in Δ4a and Δ4b mutants indicate frameshift mutations in 4a and 4b sequence caused by the deletions. (B) Growth kinetics of Δ4a and Δ4b deletion mutants in Huh-7 cells at the indicated MOIs. Supernatants were collected at 24, 48 and 72 hpi and titrated by plaque assay. (C) NF-κB-dependent cytokine response of Huh-7 cells either mock-infected or infected with WT, Δ4ab, Δ4a or Δ4b mutants (MOI = 1 PFU/cell). At 24 hpi, mRNA levels of IL-6, IL-8 and TNF-α were quantified by RT-qPCR and compared to those in WT-infected cells, using the ΔΔCt method for calculation and HMBS as a reference endogenous gene. Shown are means with standard deviations, which were analyzed using an unpaired t-test against the wild-type (**, p<0.01; ***, p<0.001).

In order to determine the specific contribution of 4a and 4b proteins to the inhibition of the NF-κB response during infection, individual deletion mutants MERS-CoV-Δ4a and MERS-CoV-Δ4b were engineered (Fig 1A). The 4a and 4b genes have overlapping coding sequences and are expressed from the same subgenomic mRNA, and thus 4b is likely expressed by either internal ribosomal entry or “leaky scanning” of ribosomes [10]. The 4a deletion preserved the transcription-regulating sequences (TRS) preceding 4a gene, which comprise the conserved sequence (CS), and 26 nt downstream of the CS, including the first 22 nt of ORF 4a. In addition, the 3’ 101 nt of the 4a gene preceding ORF 4b were maintained in order to preserve translation of 4b protein, as suggested for other CoV ORFs that are translated by similar mechanisms [11]. In the case of MERS-CoV-Δ4b the 4b gene was almost entirely deleted excluding the 4a/4b overlapping sequences and the 3’ most 53 nt, in order to maintain the TRS for transcription of gene 5 (Fig 1A). MERS-CoV-Δ4a and MERS-CoV-Δ4b were recovered from plasmids pBAC-MERS^FL^-Δ4a and pBAC-MERS^FL^-Δ4b, respectively, in Huh-7 cells with titers similar to those of wild-type virus. After two passages in cell culture, the recombinant viruses were sequenced to confirm their stability. The replication kinetics of these singly mutated viruses in Huh-7 cells was not significantly different from that of the wild-type virus (Fig 1B), with only a slight decrease in titers at some time points. These results confirm that proteins 4a and 4b were not required for virus replication in cell culture [8]. The presence or absence of 4b and 4a proteins in Huh-7 cells infected with the recombinant viruses MERS-CoV-Δ4a, MERS-CoV-Δ4b and MERS-CoV-Δ4ab was confirmed by Western-blot and immunofluorescence assays. The expression levels of 4a and 4b proteins (A in S2 Fig) and their subcellular distribution (B and C in S2 Fig) were similar to those of the wild-type virus, indicating that 4a and 4b gene expression was not affected in the deletion mutants. While 4a protein showed a cytoplasmic distribution [9, 12, 13] partially colocalizing with viral dsRNA, 4b protein localized to the nucleus of infected cells, as previously described [10].

The expression of mRNAs coding for NF-κB-dependent pro-inflammatory cytokines was analyzed in Huh-7 cells infected with the deletion mutants Δ4a and Δ4b at a multiplicity of infection (MOI) of 1 PFU/cell (Fig 1C). While deletion of 4a did not have any effect on the expression of IL-6, IL-8 and TNF-α as compared to the wild-type virus, deletion of 4b significantly increased their expression to similar or even higher levels than those observed in MERS-CoV-Δ4ab, indicating that the 4b protein contributed to the repression of NF-κB-dependent gene expression.

### Engineering recombinant MERS-CoVs with mutations in 4b nuclear localization signals (NLS)

**Fig 2:**
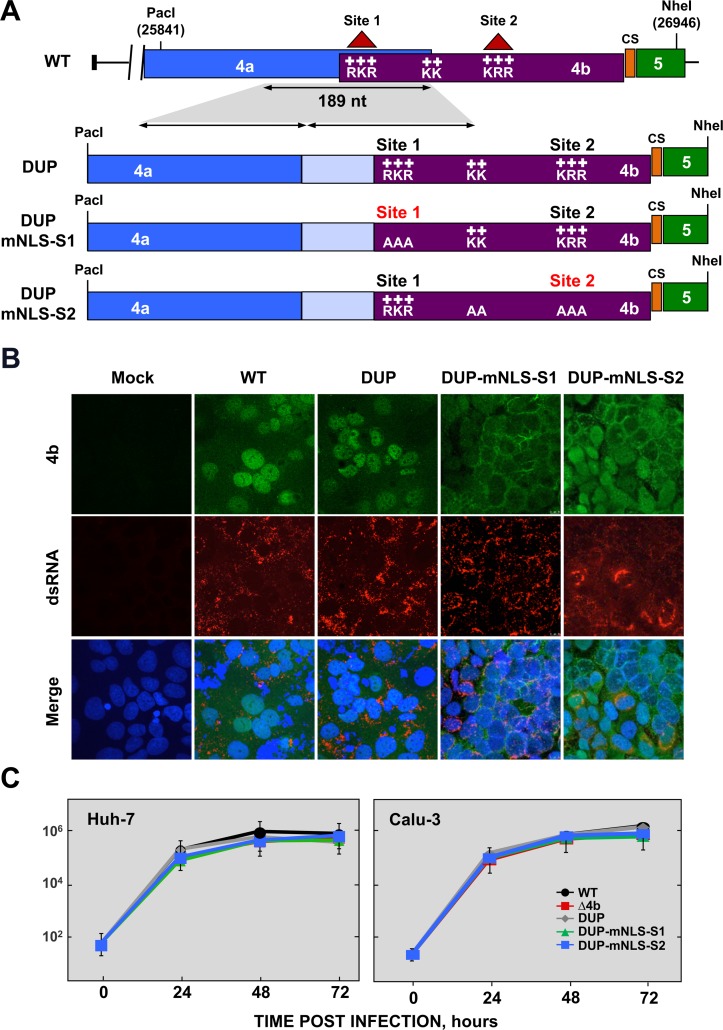
Generation of MERS-CoV-4b-NLS mutants. (A) Schematic representation of 4a and 4b overlapping sequences in MERS-CoV WT (top) and 4b-NLS mutants (bottom). Nuclear localization signals in 4b protein (NLS-S1 and NLS-S2), consisting of positively charged amino acids lysine (K) and arginine (R) are indicated. The 189 nt-sequence duplicated in 4b-NLS mutants is indicated by the shadowed area and the arrows. DUP, mutant including WT duplicated 4a-4b sequences, used as a control; DUP-mNLS-S1, mutant with 4b NLS Site 1 mutated to alanine; DUP-mNLS-S2, mutant with Site 2 and two close Lys residues mutated to alanine, as described in Materials and Methods. The light blue boxes in DUP, DUP-mNLS-S1 and DUP-mNLS-S2 mutants indicate duplicated 4a sequences. PacI and NheI restriction sites used for the assembly of infectious cDNA clones and their genomic positions (first nucleotide of the recognition sequence) are indicated. (B) Analysis by confocal microscopy of 4b subcellular localization in Huh-7 cells either mock-infected or infected with MERS-CoV-4b-NLS mutants (MOI = 0.1 PFU/cell, 24 hpi). 4b protein (green) and dsRNA (red) were detected with specific antibodies, while nuclei (blue) were stained with DAPI. (C) Growth kinetics of MERS-CoV-4b-NLS mutants in Huh-7 and Calu-3 cells at an MOI of 0.1. Supernatants were collected at 24, 48 and 72 hpi and titrated by plaque assay. Error bars represent SD.

To determine whether import of 4b protein into the nucleus was required for interference with the NF-κB pathway, MERS-CoV mutants defective in 4b nuclear localization were engineered (Fig 2A). MERS-CoV 4b protein includes a bipartite NLS consisting of two triplets of positively charged amino acids [10], named site 1 (NLS-S1) and site 2 (NLS-S2) (Fig 2A). Mutation of NLS-S1 in the context of 4b overexpression completely prevented nuclear import, while mutation of NLS-S2 only partially hindered nuclear localization of 4b [10]. Two consecutive lysine residues located between NLS-S1 and NLS-S2 might also contribute to the nuclear localization of 4b, and were included in our mutagenic analysis.

Since the 4b NLS-S1 is located within the 4a/4b overlapping sequence, its mutagenesis required the separation of 4a and 4b genes by duplicating the common region. A recombinant MERS-CoV-DUP cDNA with a 189 nt duplication including the 4a/4b overlapping sequences (88 bp) and 4a preceding sequences (101 bp), likely required for 4b translation, was generated as a control (DUP, Fig 2A). Two mutant MERS-CoV cDNAs including the 4a/4b duplication were then engineered either containing alanine mutations of NLS-S1 (RKR→AAA, MERS-CoV-DUP-mNLS-S1), or both the intermediate Lys residues and NLS-S2 (KK→AA and KRR→AAA, MERS-CoV-DUP-mNLS-S2). Recombinant viruses were rescued from infectious cDNAs in Huh-7 cells and the expression of 4a and 4b proteins was characterized. No significant differences in the expression levels of 4a and 4b proteins in Huh-7 cells were observed by Western-blot analysis, as compared to the wild-type virus (A in S3 Fig), confirming that genome engineering of 4b NLS mutants did not affect virus gene expression. Accordingly, the cytoplasmic distribution of 4a protein observed in MERS-CoV-wild-type-infected cells was not affected in the NLS mutants (B in S3 Fig). Analysis by confocal microscopy of 4b subcellular localization (Fig 2B) confirmed that 4b protein was mostly detected in the nucleus in MERS-CoV-DUP-infected cells, as observed in cells infected with wild-type virus. In contrast, mutation of 4b NLS-S1 in MERS-CoV-DUP-mNLS-S1 prevented the nuclear localization of 4b, which was mainly restricted to the cytoplasm. Mutation of NLS-S2 in MERS-CoV-DUP-mNLS-S2 only partially prevented the nuclear import of 4b, with protein detected in both the nucleus and the cytoplasm. These results demonstrated that in the context of infection, 4b NLS-S1 was essential for nuclear localization, while NLS-S2 had only a partial effect on 4b nuclear import, as described in 4b overexpression experiments [10]. The growth kinetics of MERS-CoV NLS mutants in Huh-7 and Calu-3 (a bronchial epithelial cell line) cells (Fig 2C and C in S3 Fig) confirmed that neither the duplication of 4a-4b sequences nor the mutation of 4b NLS-S1 or NLS-S2 affected viral replication as compared to the wild-type virus. This indicates that the nuclear localization of 4b protein during infection did not have a significant impact on virus replication in cell culture.

### The NLS sequences of 4b are required for the repression of NF-κB-dependent pro-inflammatory cytokine expression during infection

**Fig 3:**
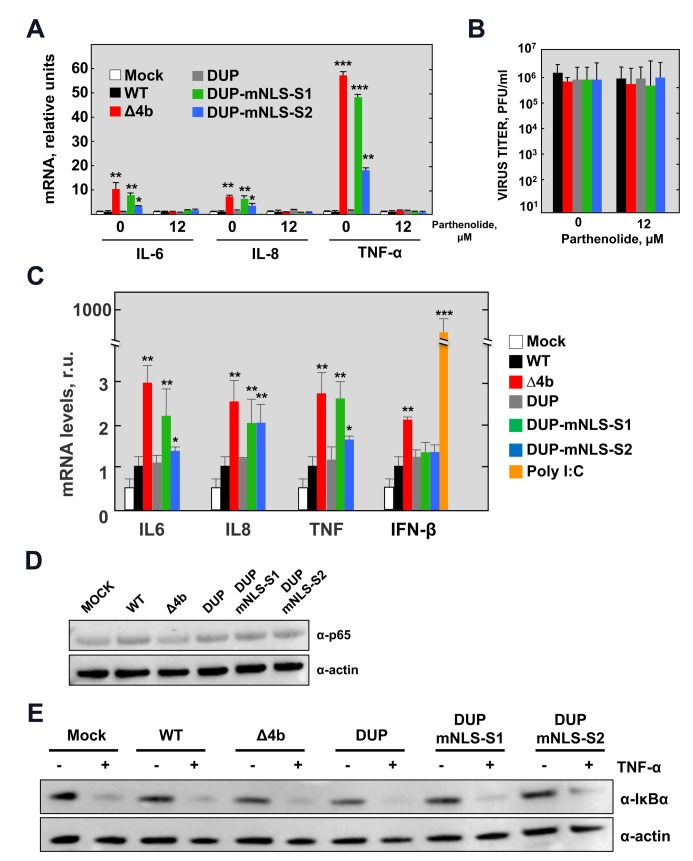
NF-κB-dependent cytokine response during infection with MERS-CoV-4b-NLS mutants (MOI = 1 PFU/cell, 24 hpi). (A) The mRNA expression levels of IL-6, IL-8 and TNF-α were quantified by RT-qPCR in Huh-7 cells either mock-treated or treated with the NF-κB inhibitor parthenolide (12 μM), as described in Fig 1. Error bars represent SD. (B) Viral titers in the supernatant of Huh-7 cells infected with MERS-CoV-4b-NLS mutants either mock-treated or treated with parthenolide. (C) The mRNA expression levels of IL6, IL8, TNF-α and IFNB1 were quantified by RT-qPCR in Calu-3 cells infected with MERS-CoV-4b-NLS mutants as described in S1 Fig. (D) Analysis by Western-blot of NF-κB p65 levels at 24 hpi in Huh-7 cells infected with 4b-NLS mutants (MOI = 1 PFU/cell). (E) Analysis by Western-blot of TNF-α induced IκBα degradation in Huh-7 cells either mock-infected or infected with MERS.CoV-4b-NLS mutants (MOI = 1 PFU/cell). At 14 hpi, cell supernatant was replaced by fresh medium containing TNF-α (50 ng/ml). After 30 min treatment, cell lysates were prepared for immunoblotting with anti IκBα antibody. Actin was used as a loading control. Shown are means with standard deviations, which were analyzed using an unpaired t-test against the wild-type (*, p<0.1**; p<0.01; ***, p<0.001).

To evaluate the relevance of 4b nuclear localization in the antagonism of the innate immune response during infection, Huh-7 and Calu-3 cells were infected with 4b-NLS mutants at a MOI of 1 PFU/cell. The expression of NF-κB-mediated pro-inflammatory cytokines IL-6, IL-8 and TNF-α was analyzed by RT-qPCR at 24 hpi (Fig 3A–3C). Mutation of NLS-S1 led to significantly elevated expression levels of NF-κB dependent pro-inflammatory cytokines, similar to those induced by MERS-CoV-Δ4b. Mutation of NLS-S2, leading to a partially nuclear 4b protein, increased the expression of pro-inflammatory cytokines to intermediate levels as compared to the NLS-S1 mutation. These results indicated that nuclear translocation of 4b was required to inhibit the expression of NF-κB dependent pro-inflammatory cytokines both in Huh-7 and Calu-3 cells, suggesting that this effect might be relevant in the natural infection. Thus, MERS-CoV infection induced an NF-κB-dependent response either in the absence of 4b or in the presence of 4b proteins deficient in nuclear localization.

MERS-CoV infection of Calu-3 cells in the absence of 4b protein only led to a two-fold increase in IFN-β levels, while no difference in IFN-β levels was observed in Huh-7 cells. This is in contrast to the high expression levels induced by the positive control poly(I:C) (Fig 3C). 4b NLS mutants did not induce IFN-β production in Calu-3 cells, suggesting a limited contribution of 4b protein to the suppression of the IFN- β response and confirming the idea that multiple viral proteins participate in the antagonism of the innate immune response.

### Effect of 4b-NLS mutations in the NF-κB signaling pathway during MERS-CoV infection

To confirm that the pro-inflammatory response induced by MERS-CoV in the absence of nuclear 4b protein was mediated by NF-κB, Huh-7 cells were treated with the NF-κB inhibitor parthenolide [14] at the same time cells were infected. Parthenolide treatment inhibited the expression of NF-κB dependent pro-inflammatory cytokines induced by 4b mutant viruses without affecting virus production (Fig 3A and 3B). This confirmed that NF-κB mediated the pro-inflammatory response observed in 4b-mutant virus infected cells.

To determine the step in the NF-κB signaling pathway targeted by 4b protein, the amount of NF-κB p65 subunit was first analyzed by Western-blot in cells infected with 4b-mutant MERS-CoVs. No differences in p65 levels were observed among MERS-CoV mutants regardless of the NF-κB-mediated response (Fig 3D). Since activation of NF-κB requires the degradation of the inhibitor IκBα, levels of IκBα were analyzed by Western-blot in cells infected with 4b-mutant MERS-CoVs (A in S4 Fig). It was difficult to detect IκBα degradation in the context of viral infection, most likely indicating that activation of the pathway was not complete. Alternatively, activated NF-κB has been reported to rapidly induce expression of IκBα to restore NF-κB inhibition [15]. To examine the impact of viral infection on IκBα degradation, we further treated infected cells with TNF-α for different time periods at 14 hours post-infection, which is expected to induce a sustained reduction in IκBα levels. At this time point, significant levels of 4b had accumulated (B in S4 Fig), providing a scenario where we could evaluate potential inhibition of IκBα degradation by 4b. Degradation of IκBα in infected cells treated with TNF-α was associated with induction of pro-inflammatory mRNAs, thus confirming that TNF-α activated the NF-κB pathway (C in S4 Fig). Furthermore, 4b protein significantly reduced the expression levels of pro-inflammatory cytokines induced by TNF-α in infected cells as compared to infection with Δ4b virus, reinforcing the potential of 4b protein to repress the NF-κB response (C in S4 Fig). However, no differences in IκBα levels were observed in infections with Δ4b and 4b NLS-mutant MERS-CoVs after TNF-α stimulation, suggesting that the 4b protein did not inhibit the degradation of IκBα (Fig 3E and A in S4 Fig). Together, these results indicated that inhibition of the NF-κB response by the 4b protein did not occur at steps prior to the nuclear translocation of NF-κB.

### MERS-CoV 4b protein interacts with α-karyopherins

**Fig 4:**
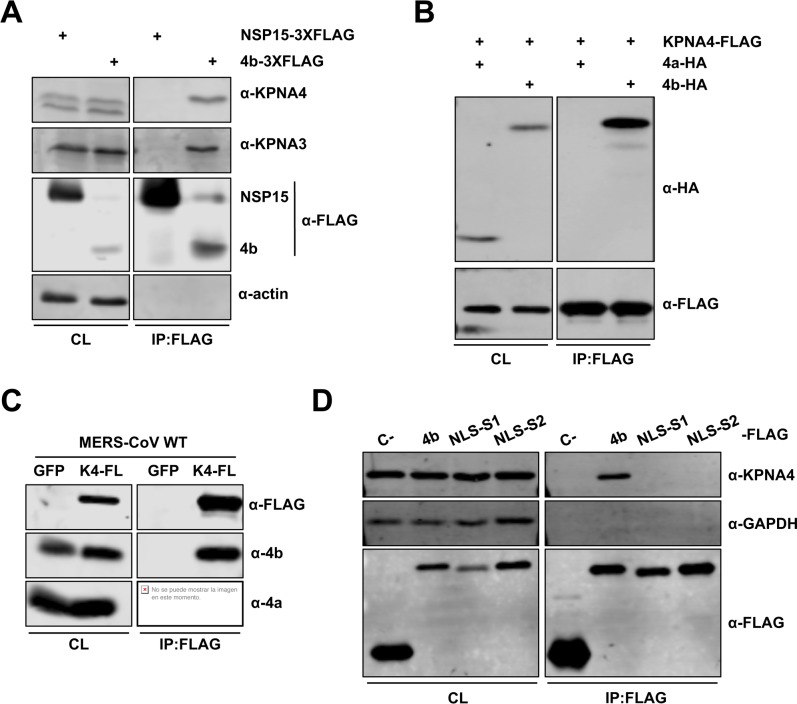
MERS-CoV 4b protein interacts with importin-α2 family karyopherins. (A-B). 4b interacts with KPNA3 and KPNA4 when expressed in isolation. Huh-7 cells were transfected with plasmids expressing NSP15-3XFLAG or 4b-3XFLAG (A) or KPNA4-FLAG and 4a-HA or 4b-HA (B). Cells were collected 48 hours later and cell lysates were immunoprecipitated with anti-FLAG monoclonal antibody. Cell lysates (CL) and eluted proteins were analyzed by immunoblotting with indicated antibodies. (C) 4b interacts with KPNA4 during infection. Huh-7 cells were transfected with plasmids expressing GFP or KPNA4-FLAG (K4-FL). 48 hours later cells were infected with MERS-CoV at an MOI of 0.1 PFU/cell. Cells were collected at 20 hpi and cell lysates were immunoprecipitated and analyzed as described above. (D) 4b requires both NLS Site 1 and Site 2 for efficient interaction with KPNA4. Huh-7 cells were transfected with plasmids expressing sMacro-3XFLAG (C-), 4b-3XFLAG (4b), or the 4b-NLS mutants 4b-mNLS-S1–3XFLAG (NLS-S1), or 4b-mNLS-S2–3XFLAG (NLS-S2). Cells were collected 48 hours later and cell lysates were immunoprecipitated and analyzed as described above. Viral protein 4a and cell proteins actin and GAPDH have been used as controls for non-specific binding to KPNA4-FLAG or to FLAG-antibody coated beads. GFP has been used as a control for 4b non-nonspecific binding to the FLAG-antibody coated beads in the absence of karyopherin protein.

In a co-immunopecipitation (Co-IP) mass spectrometry screen for 4b interacting partners, we identified several peptides corresponding to karyopherin (KPNA)-α4 and KPNA-α3 (also known as importin-α3 and importin-α4, respectively) that were immunoprecipitated with overexpressed 4b-FLAG protein but not the SARS-CoV control protein nsp15-FLAG (S5 Fig). These interactions were confirmed by reciprocal co-immunoprecipitation experiments followed by Western blotting (Fig 4A and 4B). Specifically, pull-down of 4b-FLAG from Huh-7 cells with a FLAG antibody isolated endogenous karyopherin-α4 and karyopherin-α3, but not the control protein actin (Fig 4A). Conversely, pull-down of overexpressed KPNA4-FLAG in Huh-7 cells with FLAG antibody isolated the co-expressed 4b-HA protein but not the 4a-HA protein (Fig 4B). To confirm that 4b interacts with KPNA4 in the context of infection, KPNA4-FLAG or control GFP expressing plasmids were transfected into Huh-7 cells, prior to infection with wild-type virus. At 20 hpi, the cell lysates were immunoprecipitated with FLAG antibody followed by Western-blot analysis for either endogenous 4b or 4a proteins (Fig 4C). Significant amounts of 4b but not 4a protein were isolated with KPNA4-FLAG but not GFP expressing cells, indicating a strong and specific interaction.

The nuclear translocation of most cellular proteins is mediated by α-karyopherins (or importin-αs), which directly bind the NLS within cargo proteins, and karyopherin-β (or importin-β). The latter interacts with nucleoporins to promote nuclear import [16]. Since MERS-CoV 4b protein includes a bipartite NLS and localizes to the nucleus during infection, we tested whether the bipartite NLS sequences of 4b were required for KPNA4 binding. The basic amino acids in NLS-S1 or NLS-S2 were replaced with alanine in plasmids (4b-mNLS-S1–FLAG or 4b-mNLS-S2–FLAG) and subsequently transfected into Huh-7 cells. Cell lysates were analyzed by co-immunoprecipitation with FLAG antibodies followed by Western-blot (Fig 4D). Endogenous KPNA4 did not co-immunoprecipitate with 4b proteins mutated either in NLS-S1 or NLS-S2, in contrast to wild-type 4b protein. This indicates that MERS-CoV 4b protein requires both NLS-S1 and NLS-S2 for efficient interaction with KPNA4. It also strongly suggests that 4b interacts with KPNA4 as a mechanism to enter the nucleus.

**Fig 5:**
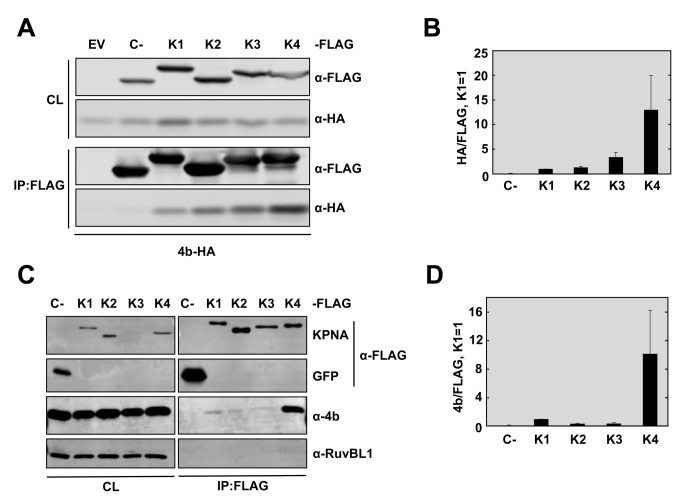
MERS-CoV 4b has higher affinity for interaction with karyopherin-α4 than other karyopherins. (A) Huh-7 cells were co-transfected with plasmids expressing 4b-HA and empty vector (EV) or Control-3XFLAG (C-) or KPNA-FLAG proteins KPNA1-FLAG (K1), KPNA2-FLAG (K2), KPNA3-FLAG (K3), or KPNA4-FLAG (K4). Cells were collected 48 hours later and cell lysates (CL) were immunoprecipitated with anti-FLAG monoclonal antibody. Cell lysates and eluted proteins were analyzed by immunoblotting with indicated antibodies. (B) Quantification of 4b-karyopherin interactions. The relative binding of 4b to each karyopherin was determined by measuring the HA signal and dividing by the FLAG signal, derived using Image Studio Software. The ratio of 4b-HA to KPNA1-FLAG was set to 1. Data represent the combined results of 2 independent experiments. Error bars represent SD. (C) Huh-7 cells were transfected with plasmids expressing GFP-3XFLAG (C-) or KPNA-FLAG proteins KPNA1-FL (K1), KPNA2-FL (K2), KPNA3-FL (K3), or KPNA4-FL (K4). 48 hours later, cells were infected with WT MERS-CoV at a MOI of 0.1 PFU/cell. Cells were collected at 20 hpi and cell lysates were immunoprecipitated with anti-FLAG monoclonal antibody and analyzed as described in (A). Cell protein RuvBL1 has been used as a control for non-specific binding to KPNA4-FLAG or to FLAG-antibody coated beads. GFP and C- have been used as controls for 4b non-nonspecific binding to the FLAG-antibody coated beads in the absence of karyopherin protein. (D) The relative binding of 4b to each karyopherin during infection was determined as described in (B). These data represent the combined results of 2 independent experiments. Error bars represent SD.

The α-karyopherins are divided into 3 sub-families. The importin-α1 subfamily, consisting of KPNA2 and KPNA7, are general importers of cargo that bear a classic NLS; the importin-α2 subfamily, consisting of KPNA4 and KPNA3, are known for their specificity for NF-κB and the regulator of chromosome condensation (RCC)-1; and finally the importin-α3 subfamily, consisting of KPNA1, KPNA5, and KPNA6, is best known for binding phosphorylated STATs as well as several Influenza A virus proteins and Ebola virus VP24 [17]. In our screen, 4b protein only pulled down importin-α2 subfamily members (S5 Fig), which are known to bind NF-κB. To directly test whether 4b preferentially interacted with importin-α2 subfamily proteins, we analyzed the ability of 4b protein to interact with α-karyopherins of each subfamily. KPNA1 (importin-α3 subfamily), KPNA2 (importin-α1 subfamily), and KPNA3 and KPNA4 (importin-α2 subfamily) were expressed as KPNA-FLAG fusion proteins in Huh-7 cells along with HA-tagged 4b protein. Cells were collected 48 hours post-transfection and cell lysates were immunoprecipitated with an anti-FLAG monoclonal antibody. The presence of KPNA and 4b protein was detected by Western-blot with anti-FLAG and anti-HA antibodies, respectively (Fig 5A). Quantification of relative binding to each karyopherin (Fig 5B) showed that 4b protein preferentially bound to KPNA4, and to a lesser extent, KPNA3. Binding of 4b protein to karyopherin-α family members during infection was assayed in a similar fashion. Huh-7 cells were transfected with plasmids expressing KPNA-FLAG fusion proteins and infected 48 hours later with wild-type MERS-CoV at an MOI of 0.1 PFU/cell. Cells were collected at 20 hpi and cell lysates were immunoprecipitated with an anti-FLAG antibody and analyzed with anti-4b and anti-FLAG antibodies (Fig 5C). In the context of infection, MERS-CoV 4b protein also preferentially bound to KPNA4 over all other karyopherins, including KPNA3 (Fig 5C and 5D). However, 4b could at least minimally bind to other KPNAs, possibly explaining the ability of 4b-mNLS-S2 to partially access the nucleus. The favorable interaction between 4b and KPNA4 is consistent with the idea that 4b prevented the nuclear import of NF-κB by binding to KPNA4 [18].

### MERS-CoV 4b protein inhibits the nuclear translocation of NF-κB in the context of infection

**Fig 6:**
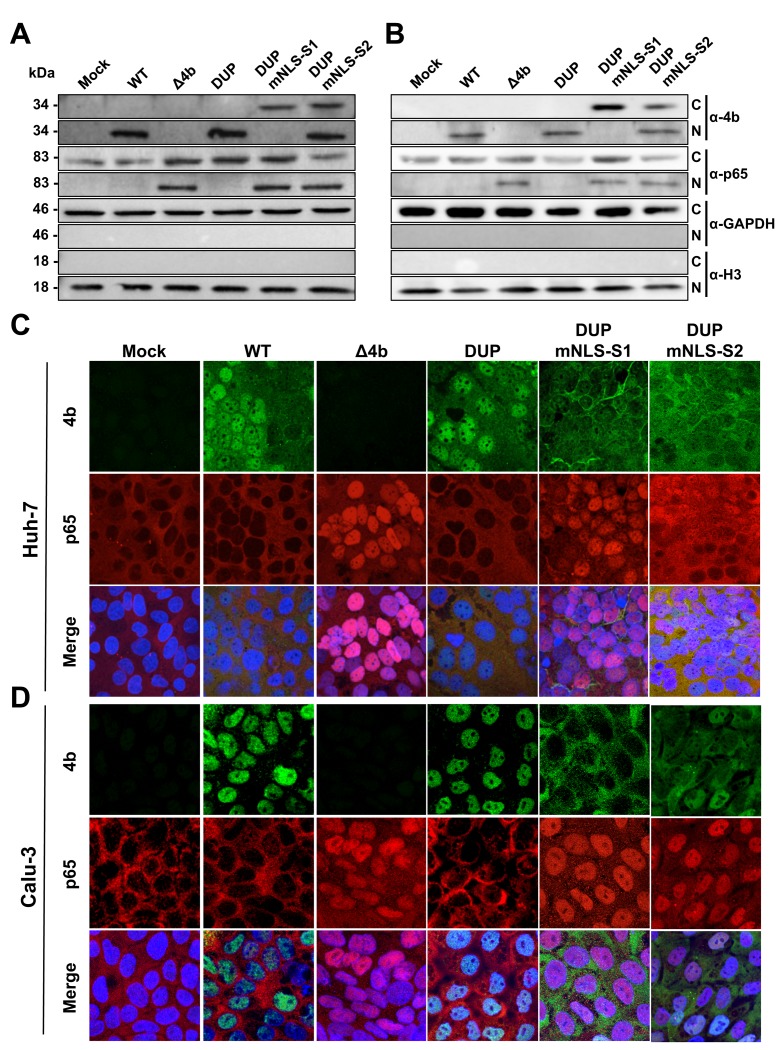
Subcellular localization of NF-κB during infection with MERS-CoV 4b-NLS mutants. Huh-7 (A) or Calu-3 (B) cells were mock-infected or infected (MOI = 0.1 PFU/cell) with WT, Δ4b or 4b-NLS mutants. At 18 hpi, cells were fractionated into cytoplasmic (C) and nuclear (N) fractions and analyzed by Western-blot for 4b and p65 detection. GAPDH and histone H3 were used as cytoplasmic and nuclear markers, respectively. Huh-7 (C) or Calu-3 (D) cells were infected with WT, Δ4b or 4b-NLS mutants (MOI 0.1 PFU/cell). At 24 hpi, cells were fixed and stained with antibodies against 4b (green) and p65 (red). Cell nuclei were stained with DAPI (blue).

Together, the above-mentioned results indicated that 4b protein specifically bound to KPNA4 both in the absence and in the presence of infection. Additionally, during MERS-CoV infection, the expression of pro-inflammatory cytokines was inhibited in the presence of wild-type nuclear 4b protein, in contrast to 4b cytoplasmic mutants. Therefore, we hypothesized that in infected cells, wild type 4b protein would interfere with NF-κB nuclear translocation. To test this possibility, we analyzed the nuclear-cytoplasmic distribution of 4b and the NF-κB p65 subunit at 24 hpi in Huh-7 and Calu-3 cells infected at an MOI of 0.1 PFU/cell either with wild-type MERS-CoV or with MERS-CoV recombinant viruses expressing cytoplasmic 4b-NLS mutant proteins. Western blot analysis of nuclear and cytoplasmic fractions and confocal microscopy analysis showed that during infection with the control viruses, MERS-CoV-wild-type or DUP, 4b protein mainly localized to the nucleus while most of the p65 protein was detected in the cytoplasm (Fig 6A–6C and S6 Fig). In contrast, in infections either with MERS-CoV-Δ4b or MERS-CoV-DUP-mNLS-S1, p65 was detected both in the nucleus and the cytoplasm. In MERS-CoV-DUP-mNLS-S2 infection, the dual distribution of 4b protein between the nucleus and the cytoplasm was associated with the presence of p65 in both cellular compartments. This observation suggested that partial access to the nucleus of 4b-mNLS-S2 was enough to allow a significant increase in p65 nuclear import as compared to that observed in the presence of nuclear wild-type 4b protein. Together, these results indicated that 4b protein with an intact NLS prevented nuclear import of NF-κB in Huh-7 cells and in the physiologically relevant Calu-3 cells, suggesting that this mechanism might also be critical in a natural lung infection.

**Fig 7:**
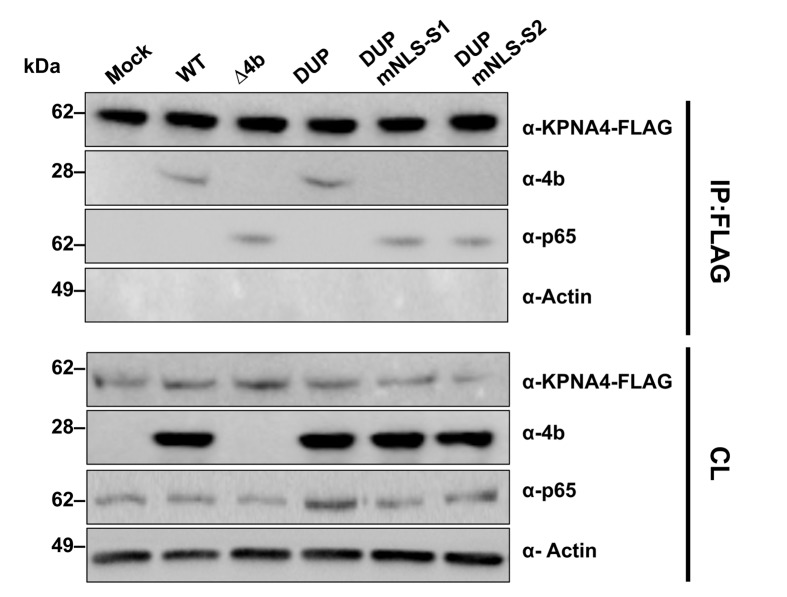
Interference of MERS-CoV 4b protein in NF-κB-karyopherin 4 binding during infection. Huh-7 cells were transfected with a plasmid encoding karyopherin 4 (KPNA4)-FLAG. At 24 hpt, cells were infected (MOI 0.1 PFU/cell) with WT, Δ4b or 4b-NLS mutants. At 20 hpi, cells lysates were immunoprecipitated with anti-FLAG antibodies. Cell lysates (CL) and eluted proteins were analyzed by immunoblotting with indicated antibodies.

Since KPNA4 binds both p65 [18] and 4b (Fig 4D) proteins through their NLS sequences, it is conceivable that 4b and p65 compete for binding to the same site in KPNA4. To address this question, co-IP experiments with FLAG antibodies in Huh-7 cells transfected with a KPNA4-FLAG plasmid and infected with MERS-CoVs expressing 4b-NLS mutant proteins were performed. These results confirmed that binding of KPNA4 to 4b protein was also NLS-dependent in the context of infection (Fig 7). Interestingly, in wild-type MERS-CoV-infected cells, immunoprecipitation of KPNA4 did not pull-down p65 while it did it in Δ4b and 4b NLS mutant virus infected cells. Together, these results strongly suggest that 4b interacts with KPNA4 in a NLS-dependent manner as a mechanism to gain entry into the nucleus, leading to the inhibition of p65 nuclear import by KPNA4.

## Discussion

MERS-CoV strongly represses the innate immune response [1], although the specific factors that contribute to this repression during infection are unknown. To date, all studies involving MERS-CoV proteins and their interactions with the innate immune response have been done with viral proteins expressed in isolation [10, 12, 13, 19, 20]. These studies can give a glimpse into the mechanisms used by the virus to block interferon and other innate immune factors. However, proteins expressed in isolation may not function as they do in the context of an infection, due to excessive protein production, improper localization, or the presence/absence of specific cellular or viral proteins [21]. Previous reports indicated that when expressed in isolation, 4b blocked IFN expression induced by Sendai virus (SeV) or by overexpression of cellular factors that function in the IFN-induction pathways [20]. Inhibition of IRF-3 or IRF-7-induced IFN production required the NLS sequences of 4b, whereas deletion of 4b NLS did not affect IFN expression induced by other factors such as MDA5, MAVS, IKKε and TBK-1. These results suggested that 4b could inhibit the induction of IFN-β in both the nucleus and the cytoplasm. Here, we show that MERS-CoV-Δ4b infection did not induce IFN in Huh-7 cells, while a limited IFN response was observed in Calu-3 cells, suggesting that additional mechanisms independent of 4b expression are used by MERS-CoV to inhibit IFN production [3, 22, 23].

CoV accessory proteins are not required for viral replication in cell culture although potentially contribute to pathogenesis *in vivo*, possibly by interfering with the innate immune response. In contrast to essential viral proteins, which are conserved across the CoV family, the accessory proteins show high diversity among CoV genera. In particular, the 4b protein is specific for MERS-CoV and does not show significant homology to other CoV accessory proteins, with the exception of two related bat CoVs HKU4 and HKU5 (homology at the amino acid level lower than 30%) and hedgehog CoVs included within the same lineage C of the genus *Betacoronavirus* [24–26]. No significant homology to other viral or mammalian proteins was found in protein databases.

**Fig 8:**
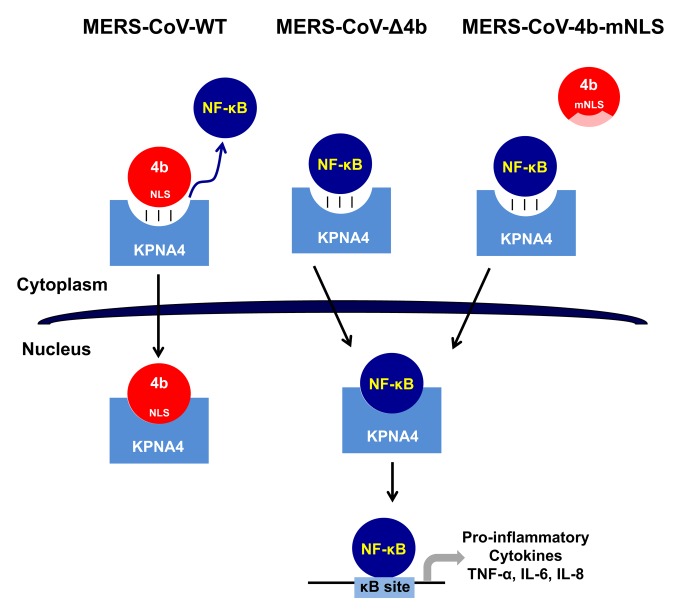
Proposed mechanism of 4b action in MERS-CoV infection. In WT MERS-CoV infection, 4b protein with an intact NLS binds KPNA4 for nuclear translocation thereby outcompeting NF-κB binding to KPNA4, which retains NF-κB in the cytoplasm and inhibits the expression of NF-κB-dependent pro-inflammatory cytokines. In contrast, in the absence of 4b (MERS-CoV-Δ4b) or in the presence of cytoplasmic 4b NLS-mutants (MERS-CoV-4b-mNLS), which do not bind KPNA4, NF-κB is imported into the nucleus by KPNA4, where it binds to DNA κB sites to promote the expression of pro-inflammatory cytokines (TNF-α, IL-6 and IL-8).

Here we demonstrate the role of the MERS-CoV accessory protein 4b during infection, using recombinant viruses that either completely delete 4b or alter its ability to traffic to the nucleus. We found that 4b was required to prevent a robust NF-κB dependent response during infection of both Huh-7 and Calu-3 cells (S1 Fig and Fig 1C). This function required the NLS sequences of 4b, as opposed to a previous report [10], in which overexpressed 4b proteins lacking the NLS were still able to inhibit IFN-β induction and to a lesser extent NF-κB signaling. Importantly, we found that the nuclear localization of 4b was associated with the cytoplasmic retention of NF-κB (Fig 6) and consequently, with the repression of NF-κB-mediated expression of pro-inflammatory cytokines (Fig 3A and 3C). We also found that both in isolation and during infection, 4b interacted with α-karyopherin proteins in an NLS-dependent manner. Interestingly, 4b had a strong preference for binding karyopherin-α4, which is also known to translocate the NF-κB protein into the nucleus [18]. Interestingly, 4b binding to KPNA4 during infection inhibited its interaction with NF-κB-p65 subunit (Fig 7). Thereby we propose a model where 4b outcompetes NF-κB for KPNA4 binding and translocation into the nucleus (Fig 8). Alternatively, other functions of 4b or additional MERS-CoV proteins might also contribute to the NF-κB antagonism, as described for other coronaviruses, which include multiple proteins that antagonize the host IFN and NF-κB responses [22, 27].

It has been reported that MERS-CoV 4b has phosphodiesterase (PDE) activity and antagonizes OAS-RNase L pathway by enzymatically degrading 2’,5’-oligoadenylate (2-5A), the activators of RNase L [28]. RNase L is an effector of the IFN-induced antiviral response and cleaves viral and host single-stranded RNA, leading to cell death and preventing viral replication and spread. Nuclear localization of 4b is exceptional among the other known viral PDEs, found in the cytoplasm. However, in the context of MHV infection, minor expression levels of MERS-CoV 4b in the cytoplasm are sufficient to prevent RNase L activation and, in fact, removal of the NLS did not improve RNase L antagonism. The relevance of 4b PDE activity to promote MERS-CoV replication requires further confirmation in appropriate cell types. Our results here and this previous study [28] suggest that 4b protein would provide MERS-CoV with multiple mechanisms for evasion of innate immunity, such as the repression of the NF-κB-mediated response and the inhibition of RNase L activation.

Although CoVs are cytoplasmic viruses, they do interact with the nucleus in diverse ways including relocation to the nucleus of specific viral proteins and the disruption of host nuclear transport [29]. This interaction can be beneficial either for viral replication or for the antagonism of host antiviral response. Some CoV genus-specific proteins, such as MERS-CoV 4b and SARS-CoV 3b, localize to the nucleus [10, 12, 20, 30], although their precise effect on host nuclear processes is still unknown. The nucleocapsid (N) and nsp1 proteins have also been detected in the nucleus, although this is not a conserved property in all CoVs. N protein nuclear localization has been related to cell cycle arrest and recruitment to the cytoplasm of nuclear proteins involved in viral RNA synthesis [29, 31]. The presence in the nucleus of MERS-CoV nsp1 has been involved in the selective inhibition of nuclear-transcribed mRNA expression [32], while porcine epidemic diarrhea virus (PEDV) nsp1 mediates the degradation of CREB-binding protein in the nucleus, thus suppressing IFN I production [33]. CoV nsp1 and SARS-CoV ORF 3b proteins are sufficiently small to diffuse passively through the nuclear pore complex [30, 32]. In contrast, nuclear import of larger proteins such as N and MERS-CoV 4b require host transporters and is mediated by NLS sequences [34].

Interference with the nuclear import machinery is a common viral strategy to evade the innate immune response. Viruses have evolved different mechanisms to subvert nucleo-cytoplasmic transport by targeting soluble transport receptors such as karyopherins as well as components of the nuclear pore complexes (NPC). Viruses with a nuclear stage, such as DNA viruses and some RNA viruses, promote nuclear import of viral genome and proteins by altering the NPC [35], which leads to a general inhibition of host nucleo-cytoplasmic trafficking. Herpes simplex virus ICP27 protein interacts with the nucleoporin Nup62, an structural component of the NPC, leading to the inhibition of nucleo-cytoplasmic transport pathways mediated by transportin or importins [36]. Similarly, the Epstein-Barr virus (EBV) BGLF4 kinase interacts with the nucleoporins Nup62 and Nup153 inhibiting canonical NLS-mediated nuclear import while promoting the nuclear import of non-NLS-containing EBV lytic proteins [37].

Cytoplasmic viruses also share similar strategies to inhibit nuclear import of signals required for host immune defenses. SARS-CoV ORF6 retains KPNA2 and karyopherin-β in the cytoplasm, thus preventing STAT1 nuclear import and antagonizing IFN signaling pathways [38–40]. A similar mechanism of inhibition has also been described for Ebola virus VP24 [41]. Enterovirus 71 (EV71) infection suppresses IFN responses by inhibiting STAT1/2 nuclear transport through the induction of caspase-3-dependent degradation of KPNA1 [42]. Poliovirus and rhinovirus infection induce proteolysis of the NPC components Nup153 and p62, leading to a general inhibition of nuclear import [43, 44]. The association of viral proteins with the host nuclear import pathway confirms its relevance in virus-host interactions, regulating innate immunity and cellular responses to infection.

NF-κB is a critical factor for the up regulation of innate immunity and inflammation in response to viral infections [4]. Therefore, viruses in diverse families have evolved a variety of mechanisms to target nuclear transport of the NF-κB complex and thereby inhibit its activation. The nucleocapsid protein of Hantaan virus inhibits NF-κB activation by limiting p65 nuclear translocation, as shown in overexpression experiments [45], presumably by its interaction with importin-α through a putative NLS. Japanese encephalitis virus NS5 competitively inhibits NF-κB nuclear translocation by binding to KPNA2, 3 and 4, presumably through two NLSs within NS5, which surprisingly does not accumulate in the nucleus [46]. Alternatively, viral proteins such as Herpes simplex virus 1 UL24 inhibit the NF-κB pathway by binding p65 and p50 subunits and preventing their nuclear translocation [47].

In CoVs, there is also evidence supporting the relevance of the NF-κB pathway in pathogenesis. Increased NF-κB activation is a determinant of the exacerbated lung inflammatory pathology caused by SARS-CoV infection [6]. Consequently, inhibition of NF-κB signaling significantly reduced pulmonary inflammation and increased survival of SARS-CoV-infected mice [5]. Dual regulation of NF-κB signaling during HCoV-229E infection has been described, consisting in the simultaneous induction of the NF-κB pathway, which has a pro-viral effect, and the restriction of the NF-κB response to limit transcription of pro-inflammatory and other potentially antiviral host cell genes [48]. Here, our results with MERS-CoV 4b protein are compatible with this concept of repression of the NF-κB-mediated pro-inflammatory response during CoV infection.

Viral antagonists of the innate immune response contribute to the pathogenic potential of coronaviruses *in vivo* [27, 49]. In particular, inhibition of nuclear import by SARS-CoV ORF6 has been shown to mediate pleiotropic alterations in host gene expression during infection [50] that enhance viral pathogenesis *in vivo* [51]. In addition, MERS-CoV 4b protein expressed in mice by chimeric MHV viruses acted as an RNase L antagonist contributing to pathogenesis *in vivo* [28]. From this study and from the results reported here, it might be speculated that deletion of MERS-CoV 4b protein would enhance the host antiviral response leading to virus attenuation *in vivo*. However, the possibility that the increased NF-κB-response induced by 4b deletion could result in lung inflammatory pathology [5] cannot be excluded until *in vivo* studies are performed.

Humanized mice (hDPP4-KI) infected with mouse-adapted MERS-CoV develop lethal disease and will be useful for determining the role of the 4b protein in pathogenesis. Human and mouse KPNA4 proteins share 99% amino acid identity, including binding sites of NLSs present in NF-κB p65 and p50 subunits [18]. Consequently, the interaction between MERS-CoV 4b and human KPNA4 reported here is expected to function similarly in mouse cells, inhibiting NF-κB nuclear translocation in the MERS-CoV mouse model.

In one study, no differences in lethality were observed in human DPP4 knockin mice infected with mouse adapted MERS-CoV isolates differing in the presence or absence of ORF3-4a-4b accessory genes in addition to other non-synonymous mutations [52]. In a second study, two adapted viral clones with large deletions in ORF4b, in addition to other missense mutations throughout the genome, caused increased lung inflammation and mouse weight losses [53]. However, none of these studies used congenic strains of virus solely differing in 4b expression. Infection with such strains will provide insight into the role of 4b in the context of severe infection.

## Materials and methods

### Cells and viruses

Baby hamster kidney cells (BHK-21) were obtained from American Type Culture Collection (ATCC CCL-10). Human liver-derived Huh-7 cells were kindly provided by R. Bartenschlager (University of Heidelberg, Germany). Cells were grown in BioWhittaker (Lonza) or Dulbecco’s modified Eagle medium (DMEM) with 2% glutamine, 1% non-essential amino acids (Sigma) and 5% (BHK-21) or 8% (Huh-7) fetal bovine serum (FBS) (HyClone, ThermoFisher). The bronchial epithelial cell line Calu-3 2B4 [54] was kindly provided by CT Tseng (University of Texas Medical Branch, USA). Calu-3 cells were grown in BioWhittaker (Lonza) 1 g/L glucose medium with 2% glutamine, 1% non-essential amino acids (Sigma) and 15% fetal bovine serum (FBS) (HyClone, ThermoFisher).

Virus titers were determined on Huh-7 cells following standard procedures and using closed flasks or plates sealed in plastic bags. For plaque assays, infected cells were overlaid with DMEM containing 0.6% low-melting agarose and 2% FBS, and at 72 hpi, cells were fixed with 10% formaldehyde and stained with 0.1% crystal violet. All work with MERS-CoV infectious viruses was performed in biosafety level 3 facilities at CNB-CSIC or the University of Iowa according to the guidelines set forth by each institution.

### Bacteria strain and plasmids

*E*. *coli* DH10B (Gibco/BRL) cells were transformed by electroporation using a MicroPulser unit (Bio-Rad) according to the manufacturer’s instructions and grown on antibiotic-selective LB medium.

MERS-CoV ORF4a-HA and ORF4b-HA nucleotide sequences were synthesized and cloned into pUC57 (GenScript). They were then PCR amplified, restriction digested and ligated into the pLKO plasmid. A pLKO ORF4b-3XFLAG plasmid was made by creating a PCR product replacing the HA tag with a 3XFLAG tag. This PCR product was restriction digested and ligated back into the pLKO plasmid. The resulting constructs were confirmed by restriction digest, PCR, and direct sequencing. The sMacro and GFP pcDNA3 plasmids were previously described [55]. The NSP15-3XFLAG plasmid was a generous gift of Michael Buchmeier (University of California-Irvine), and the KPNA-FLAG plasmids were a generous gift of Megan Shaw (Mount Sinai Medical Center). Cells were transfected using Polyjet (Amgen) or Lipofectamine 2000 (Fisher Scientific) as per the manufacturer’s instructions.

### Construction of deletion mutants MERS-CoV-Δ4a and MERS-CoV-Δ4b

Genes 4a and 4b were deleted from the MERS-CoV infectious cDNA clone by PCR-directed mutagenesis. The plasmid pBAC-MERS^FL^ was used as the template for amplification of overlapping PCR fragments with oligonucleotides MERS-del4a-26022-VS (5′-CTATGGATTACGTGTCTCTGCTTACAGCTATCCTTTGCTGGTTATACTGAATCT-3′) and MERS-del4a-26022-RS (5’-AGATTCAGTATAACCAGCAAAGGATAGCTGTAAGCAGAGACACGTAATCCATAG -3’) for 4a deletion or MERS-del4b-26811-VS (5’-GCGAGGAAGAGGAGCCATTCTCCAACTAAGTAATAAACAAATTGTTCATTCTTATCCC-3’) and MERS-del4b-26811-RS (5’-GGGATAAGAATGAACAATTTGTTTATTACTTAGTTGGAGAATGGCTCCTCTTCCTCGC -3’) for 4b deletion. The final PCR product was amplified with external oligonucleotides SA25412-VS (5’-CTGCACTGGTTGTGGCAC-3’) and MERS-26970-Nhe RS (5′ GCTAAAGCAGCTACATAGCCGCTAGCAGGAATG 3′; NheI is underlined), digested with PacI and NheI, and cloned into the same restriction sites of pBAC-MERS-3′ [8]. The PacI-RsrII digestion products from plasmids pBAC-MERS-3′-Δ4a and pBAC-MERS-3′-Δ4b were cloned into the same sites of pBAC-MERS^FL^ (Fig 2A). pBAC-MERS^FL^-Δ4a included a deletion from nucleotides 25,874 to 25,991 of the MERS-CoV genome, while the deletion in pBAC-MERS^FL^-Δ4b extended from nucleotides 26,183 to 26,781. For all constructs, sequences were checked after cloning by sequencing. Recombinant BACs were isolated and purified using a large-construct kit (Qiagen), following the manufacturer’s specifications.

### Construction of recombinant MERS-CoV genomes with mutated 4b NLSs

The complete sequence of 4a and 4b genes in the MERS-CoV infectious cDNA [8] was replaced with chemically synthesized fragments (GeneArt, Invitrogen) introduced into PacI-NheI sites (nt 25841 to 26945), including either the duplication of 4a/4b region alone or in combination with NLS mutations. The resulting pBAC-MERS-CoV-DUP contained a duplication of the last 189 nt of 4a gene, including the 4a/4b overlapping sequence and the preceding 4a 101 nt. Furthermore, three silent point mutations were introduced into 4a gene (T26094C, T26109C and A26112C) to prevent premature translation initiations of 4b from the sgmRNA 4ab. pBAC-MERS-DUP-mNLS-S1 included the 4b NLS-S1 (aa 22–24) mutated to alanine (RKR→ AAA) in a sequence context identical to the control pBAC-MERS-CoV-DUP, while pBAC-MERS-DUP-mNLS-S2 included alanine mutations in 4b NLS-S2 (aa 36–38) and two intermediate Lys residues, (aa 30–31) (KRR → AAA and KK → AA, respectively). The genetic integrity of the cDNAs was verified by restriction analysis and sequencing.

### Recovery of recombinant viruses from the cDNA clones

To recover infectious viruses, BHK-21 cells were transfected with the infectious cDNA clones using Lipofectamine 2000 (Invitrogen) according to the manufacturer’s specifications. At 6 h post-transfection, cells were trypsinized, plated over a confluent monolayer of Huh-7 cells, and incubated at 37°C for 72 h. The cell supernatants were harvested and passaged once on fresh Huh-7 cells and then virus titers were determined. The genetic stability of rescued viruses was verified by sequencing the genomic region comprising the 4a and 4b genes using RT-PCR.

### Growth kinetics of recombinant MERS-CoVs

Confluent monolayers of Huh-7 or Calu-3 cells were infected at a MOI of 0.1 and 0.001 PFU/cell. Cell supernatants were collected at 24, 48 and 72 hpi and virus titers were determined as described above.

### Analysis of gene expression by RT-qPCR

Total intracellular RNA was extracted from transfected or infected cells with the RNeasy miniprep kit (Qiagen) according to the manufacturer’s specifications. Total cDNA was synthesized with random hexamers from 100 ng of total RNA using a High-Capacity cDNA reverse transcription kit (Invitrogen). For qPCR assays, specific TaqMan assays (Apply Biosystems) for TNF-α (Hs99999043_m1); IL-6 (Hs00985641_m1); IL-8 (Hs99999034_m1); IFN-β (Hs02621180_s1) and hydroxymethylbilane synthase (HMBS, Hs00609297_m1, as an endogenous control) were used. MERS-CoV genomic RNA (gRNA) synthesis was analyzed using a custom TaqMan assay as described [8]. Data acquisition, analysis and normalization were performed as described [8]. Two micrograms of polyinosinic-polycytidylic acid [Poly(I:C)] were used for reverse transfection of cells with 12 μg of Lipofectamine 2000 (Invitrogen), as recommended by the manufacturer. The relative quantifications were performed using the 2^-ΔΔCt^ method [56].

### Generation of polyclonal antisera specific for MERS-CoV 4a and 4b proteins

Rabbit polyclonal antisera (pAb) specific for MERS-CoV 4a and 4b proteins were ordered from BioGenes (Germany). In brief, rabbits were immunized with synthetic peptides PDGCSLYLRHSSLFAQSEEEEPFSN (25 C-terminal residues of protein 4a) and SIRSSNQGNKQIVHSYPILHHPGF (24 C-terminal residues of protein 4b, as described) [10], according to standard protocols. The specificity of the antisera was assessed by Western-blot and immunofluorescence microscopy of MERS-CoV-infected Huh-7 cells, with pre-immune sera and mock-infected cells as negative controls.

### Immunoprecipitation

At either 48 hours post-transfection or 20 hours post-infection, Huh-7 cells were collected and pelleted by low speed centrifugation prior to lysis. Cell pellets were lysed with IP buffer (0.5–2% NP-40, 300 mM NaCl, 5% glycerol, and 50 mM Tris pH 8.0), with protease/phosphatase inhibitor cocktails (Roche), PMSF, and a universal nuclease (ThermoFisher Scientific) for 1 hour at 4ºC. Next, nuclei were pelleted by centrifugation (16,000 x g for 15 min at 4ºC). One aliquot of cell lysate was saved as the input control and boiled in SDS sample buffer in the presence of sample reducing agent. Supernatants were then applied to FLAG antibody conjugated magnetic beads (Sigma Aldrich #M8823) and were mixed for 2 hours-overnight at 4ºC. Beads were washed with TBS-Tween before elution with FLAG peptide (1 μg/ml) for 1 hour and then boiled in SDS sample buffer in the presence of sample reducing agent.

For mass spectrometry analysis, proteins precipitated by anti-FLAG antibody were resolved on a NuPAGE 4–12% gradient gel (Novex) and subsequently stained using a Silver Stain kit (Pierce) according to the manufacturer’s instructions. Protein bands unique to the 4b-3XFLAG transfected sample were extracted. In addition, gel bands from the NSP15-3XFLAG sample with migrating positions corresponding to those of 4b-3XFLAG specific bands were also extracted as negative controls. Extracted gel samples were submitted to the Keck Mass Spectrometry and Proteomics Facility (School of Medicine, Yale University) for liquid chromatography (LC)-mass spectrometry analysis for protein identification.

### Nuclear and cytoplasmic fractionation

Whole protein extracts were prepared from Huh-7 or Calu-3 cells in loading buffer [0.1 M Tris-HCl, pH 6.8; 20% glycerol; 4% w/v sodium dodecyl-sulfate (SDS); 0.2% bromophenol blue and 0.05% β-mercaptoethanol]. Nuclear and cytoplasmic fractions of Huh-7 or Calu-3 cells were prepared using a protocol adapted from [57]. Briefly, cell monolayers were washed three times with PBS, collected and resuspended in lysis buffer [0.1% IGEPAL CA-630 (Sigma-Aldrich) in PBS]. Extracts were centrifuged at 1000 x g for 1 min to recover the cytoplasmic fraction in the supernatant and the nuclei in the pellet. The cytosolic fraction was removed and mixed with Laemmli sample buffer 1:1, while the pellet was resuspended in PBS with 0.1% IGEPAL and centrifuged at 1000 x g for 1 min. The supernatant was discarded and the pellet, designated as nuclear fraction, was treated with DNase (Roche) at 37ºC for 1 hour and then mixed 1:1 with Laemmli sample buffer.

### Western blotting

Cell extracts or IP elutions were lysed in sample buffer containing SDS, protease and phosphatase inhibitors (Roche), β-mercaptoethanol, and a universal nuclease (Fisher Scientific). Proteins were resolved on an SDS polyacrylamide gel, transferred to a polyvinylidene difluoride (PVDF) or nitrocellulose membrane, hybridized with a primary antibody, reacted with either HRP or infrared (IR) dye-conjugated secondary antibody, visualized using chemiluminescent substrate (Bio-Rad) or a Li-COR Odyssey Imager (Li-COR), and analyzed using Image Studio software. Primary antibodies used for immunoblotting included anti-FLAG monoclonal antibody (Sigma-Aldrich #F1804); anti-HA monoclonal antibody (Biolegend #901513); anti-MERS-CoV 4b polyclonal antibody and anti-MERS-CoV 4a polyclonal antibodies (this study); anti-KPNA3 and anti-KPNA4 polyclonal antibodies (Thermo Scientific #PA5-18238/PA5-21033); anti-MERS-CoV N protein [8]; anti-histone H3 (Cell Signaling #9715); anti-p65 (Cell Signaling #6956); anti-GAPDH mAb (Cell Signaling #5174). Secondary antibodies used included horseradish peroxidase-conjugated anti-rabbit or anti-mouse (Sigma #A0545/A0168) antibodies; or IR conjugated anti-rabbit or anti-mouse (Li-COR, #926-68071/926-32210) antibodies.

### Analysis of the subcellular localization of 4a, 4b and NF-κB proteins by confocal microscopy

Huh-7 or Calu-3 cells were grown to 95% confluence on sterile glass coverslips and infected with MERS-CoV at a MOI of 0.1 PFU/cell. At 24 hpi, cells were fixed and the virus was fully inactivated by incubating with 4% paraformaldehyde in PBS at RT for 45 min. Cells were permeabilized with methanol at −20°C for 10 min and blocked with 10% FBS in PBS at RT for 1 h. MERS-CoV 4a and 4b proteins were detected with the specific pAbs described above. dsRNA was detected with J2 mAb (SCICONS) and NF-κB was detected with anti-p65 (Cell Signaling #6956) diluted in PBS 5% FBS. Coverslips were washed 4 times with PBS and incubated with secondary antibodies conjugated to Alexa Fluor 488 or 594 (Invitrogen) diluted 1:500 in 5% FBS in PBS at RT for 45 min. Nuclei were stained using DAPI (4′,6-diamidino-2-phenylindole) diluted 1:200 in PBS. Finally, coverslips were mounted in ProLong Gold antifade reagent (Invitrogen) and analyzed on a Leica SP8 confocal microscope. Images were acquired with the same instrument settings and analyzed with Leica software.

## Supporting information

S1 Fig Cytokine response of Huh-7 cells infected with MERS-CoV-Δ3, Δ4ab, Δ5 and WT virus. Huh-7 cells were either transfected with 2 μg poly(I:C) or infected (MOI = 1 PFU/cell) and total RNA was collected at 16 hpt or 24 hpi, respectively. IFN-β, IL-6, IL-8 and TNF-α mRNA expression levels were quantified by RT-qPCR and related to those in WT-infected cells, using the ΔΔCt method for calculation and HMBS as a reference endogenous gene. Shown are means with standard deviations, which were analyzed using an unpaired t-test against the wild-type (ns, not significant; **, p<0.01; ***, p<0.001).
(TIF)

S2 Fig Expression and localization of 4a and 4b proteins during infection with MERS-CoV-Δ4a and Δ4b deletion mutants. Huh-7 cells were mock-infected or infected with the WT, Δ4ab, Δ4a or Δ4b deletion mutants (MOI = 0.1 PFU/cell). At 24 hpi, cell lysates were analyzed by Western blot and detected with the indicated antibodies (A). MERS-CoV protein N was used as a positive control for infection. At 24 hpi cells were fixed and stained with specific antibodies against 4a (B) or 4b (C) (green) and dsRNA (red). Cell nuclei were stained with DAPI (blue).
(TIF)

S3 Fig Characterization of MERS-CoV-4b-NLS mutants. Huh-7 cells were mock-infected or infected with the WT or 4b-NLS mutants (MOI of 0.1 PFU/cell). At 24 hpi, cell lysates were analyzed by Western blot (A) with the indicated antibodies; or cells were fixed and stained with specific antibodies (B) against 4a (green) and dsRNA (red). Cell nuclei were stained with DAPI (blue). (C) Growth kinetics of MERS-CoV-4b-NLS mutants at a MOI 0.001 PFU/cell. Supernatants were collected at 24, 48 and 72 hpi and titrated by plaque assay.
(TIF)

S4 Fig IκBα degradation kinetics and NF-κB activation during infection with 4b-NLS mutants. (A) Huh-7 cells were mock-infected or infected with WT, Δ4b or 4b-NLS mutants (MOI = 1 PFU/ml). At 14 hpi, cell supernatant was replaced by fresh medium containing TNF-α (50 ng/ml). After the indicated times of treatment, cell lysates were prepared for immunoblotting with anti-IκBα antibodies. Actin was used as a loading control. (B) Huh-7 cells were mock-infected or infected with WT virus (MOI = 1 PFU/cell). After indicated times, cell lysates were collected for Western blot analysis of 4b protein expression. N protein was used as a control. (C) Huh-7 cells were mock-infected or infected with WT or Δ4b mutant (MOI = 1 PFU/ml). At 14 hpi, cells were treated with TNF-α (50 ng/ml) for 30 min. Total RNA was extracted and mRNA expression levels of TNF-α, IL-6 and IL-8 were quantified by RT-qPCR and compared to those in untreated WT-infected cells, using the ΔΔCt method and HMBS as a reference endogenous gene. Error bars represent SD.
(TIF)

S5 Fig Mass spectrometry analysis of MERS-CoV 4b protein interacting partners. KPNA3 and KPNA4 peptides specifically identified by mass spec from 4b-FLAG Co-IP samples are listed.
(TIF)

S6 Fig Quantification of 4b and NF-κB bands from Western blots in Fig 6. Protein 4b and NF-κB band intensities in cytoplasmic or nuclear fractions of Huh-7 (A) or Calu-3 (B) cells were normalized to GAPDH or H3 levels, respectively. The normalized intensity of 4b or p65 in the mock-infected samples was set to 1. These data represent the average results of 2 independent experiments. Shown are means with standard deviations, which were analyzed using an unpaired t-test against the wild-type (**, p<0.01).
(TIF)

## References

[1] Zielecki F, Weber M, Eickmann M, Spiegelberg L, Zaki AM, Matrosovich M, et al Human cell tropism and innate immune system interactions of human respiratory coronavirus EMC compared to those of severe acute respiratory syndrome coronavirus. J Virol. 2013;87:5300–4. Epub 2013/03/02. doi: 10.1128/JVI.03496-12 ; PubMed Central PMCID: PMC3624328. 23449793 10.1128/JVI.03496-12 PMC3624328

[2] Chan RW, Chan MC, Agnihothram S, Chan LL, Kuok DI, Fong JH, et al Tropism of and innate immune responses to the novel human betacoronavirus lineage C virus in human ex vivo respiratory organ cultures. J Virol. 2013;87:6604–14. Epub 2013/04/05. doi: 10.1128/JVI.00009-13 ; PubMed Central PMCID: PMC3676115. 23552422 10.1128/JVI.00009-13 PMC3676115

[3] Kindler E, Jonsdottir HR, Muth D, Hamming OJ, Hartmann R, Rodriguez R, et al Efficient replication of the novel human betacoronavirus EMC on primary human epithelium highlights its zoonotic potential. MBio. 2013;4:e00611–12. doi: 10.1128/mBio.00611-12 ; PubMed Central PMCID: PMCPMC3573664. 23422412 10.1128/mBio.00611-12 PMC3573664

[4] Li Q, Verma IM. NF-kappaB regulation in the immune system. Nat Rev Immunol. 2002;2:725–34. doi: 10.1038/nri910 . 12360211 10.1038/nri910

[5] DeDiego ML, Nieto-Torres JL, Regla-Nava JA, Jimenez-Guardeño JM, Fernandez-Delgado R, Fett C, et al Inhibition of NF-kappaB mediated inflammation in severe acute respiratory syndome coronavirus-infected mice increases survival. J Virol. 2014;88:913–24. Epub 2013/11/08. doi: 10.1128/JVI.02576-13 . 24198408 10.1128/JVI.02576-13 PMC3911641

[6] Smits SL, de Lang A, van den Brand JM, Leijten LM, van IWF, Eijkemans MJ, et al Exacerbated innate host response to SARS-CoV in aged non-human primates. PLoS Pathog. 2010;6:e1000756 Epub 2010/02/09. doi: 10.1371/journal.ppat.1000756 ; PubMed Central PMCID: PMC2816697. 20140198 10.1371/journal.ppat.1000756 PMC2816697

[7] Ludwig S, Planz O. Influenza viruses and the NF-kappaB signaling pathway—towards a novel concept of antiviral therapy. Biol Chem. 2008;389:1307–12. doi: 10.1515/BC.2008.148 . 18713017 10.1515/BC.2008.148

[8] Almazan F, DeDiego ML, Sola I, Zuñiga S, Nieto-Torres JL, Marquez-Jurado S, et al Engineering a replication-competent, propagation-defective Middle East respiratory syndrome coronavirus as a vaccine candidate. MBio. 2013;4:e00650–13. Epub 2013/09/12. doi: 10.1128/mBio.00650-13 ; PubMed Central PMCID: PMC3774192. 24023385 10.1128/mBio.00650-13 PMC3774192

[9] Rabouw HH, Langereis MA, Knaap RC, Dalebout TJ, Canton J, Sola I, et al Middle East respiratory coronavirus accessory protein 4a inhibits PKR-mediated antiviral stress responses. PLoS Pathog. 2016;12:e1005982 doi: 10.1371/journal.ppat.1005982 ; PubMed Central PMCID: PMCPMC5081173. 27783669 10.1371/journal.ppat.1005982 PMC5081173

[10] Matthews KL, Coleman CM, van der Meer Y, Snijder EJ, Frieman MB. The ORF4b-encoded accessory proteins of Middle East respiratory syndrome coronavirus and two related bat coronaviruses localize to the nucleus and inhibit innate immune signalling. J Gen Virol. 2014;95:874–82. Epub 2014/01/21. doi: 10.1099/vir.0.062059-0 . 24443473 10.1099/vir.0.062059-0 PMC3973478

[11] Snijder EJ, Bredenbeek PJ, Dobbe JC, Thiel V, Ziebuhr J, Poon LLM, et al Unique and conserved features of genome and proteome of SARS-coronavirus, an early split-off from the coronavirus group 2 lineage. J Mol Biol. 2003;331:991–1004. 12927536 10.1016/S0022-2836(03)00865-9 PMC7159028

[12] Niemeyer D, Zillinger T, Muth D, Zielecki F, Horvath G, Suliman T, et al Middle East respiratory syndrome coronavirus accessory protein 4a is a type I interferon antagonist. J Virol. 2013;87:12489–95. Epub 2013/09/13. doi: 10.1128/JVI.01845-13 ; PubMed Central PMCID: PMC3807936. 24027320 10.1128/JVI.01845-13 PMC3807936

[13] Siu KL, Yeung ML, Kok KH, Yuen KS, Kew C, Lui PY, et al Middle East respiratory syndrome coronavirus 4a protein is a double-stranded RNA-binding protein that suppresses PACT-induced activation of RIG-I and MDA5 in innate antiviral response. J Virol. 2014;88:4866–76. Epub 2014/02/14. doi: 10.1128/JVI.03649-13 . 24522921 10.1128/JVI.03649-13 PMC3993821

[14] Hehner SP, Hofmann TG, Droge W, Schmitz ML. The antiinflammatory sesquiterpene lactone parthenolide inhibits NF-kappa B by targeting the I kappa B kinase complex. J Immunol. 1999;163:5617–23. Epub 1999/11/24. . 10553091

[15] Brown K, Park S, Kanno T, Franzoso G, Siebenlist U. Mutual regulation of the transcriptional activator NF-kappa B and its inhibitor, I kappa B-alpha. Proc Natl Acad Sci USA. 1993;90:2532–6. ; PubMed Central PMCID: PMCPMC46122. 8460169 10.1073/pnas.90.6.2532 PMC46122

[16] Freitas N, Cunha C. Mechanisms and signals for the nuclear import of proteins. Curr Genomics. 2009;10:550–7. doi: 10.2174/138920209789503941 ; PubMed Central PMCID: PMCPMC2817886. 20514217 10.2174/138920209789503941 PMC2817886

[17] Pumroy RA, Cingolani G. Diversification of importin-alpha isoforms in cellular trafficking and disease states. Biochem J. 2015;466:13–28. doi: 10.1042/BJ20141186 ; PubMed Central PMCID: PMCPMC4405237. 25656054 10.1042/BJ20141186 PMC4405237

[18] Fagerlund R, Kinnunen L, Kohler M, Julkunen I, Melen K. NF-{kappa}B is transported into the nucleus by importin {alpha}3 and importin {alpha}4. J Biol Chem. 2005;280:15942–51. doi: 10.1074/jbc.M500814200 . 15677444 10.1074/jbc.M500814200

[19] Yang Y, Zhang L, Geng H, Deng Y, Huang B, Guo Y, et al The structural and accessory proteins M, ORF 4a, ORF 4b, and ORF 5 of Middle East respiratory syndrome coronavirus (MERS-CoV) are potent interferon antagonists. Protein Cell. 2013;4:951–61. Epub 2013/12/10. doi: 10.1007/s13238-013-3096-8 . 24318862 10.1007/s13238-013-3096-8 PMC4875403

[20] Yang Y, Ye F, Zhu N, Wang W, Deng Y, Zhao Z, et al Middle East respiratory syndrome coronavirus ORF4b protein inhibits type I interferon production through both cytoplasmic and nuclear targets. Sci Rep. 2015;5:17554 doi: 10.1038/srep17554 ; PubMed Central PMCID: PMCPMC4668369. 26631542 10.1038/srep17554 PMC4668369

[21] Versteeg GA, Garcia-Sastre A. Viral tricks to grid-lock the type I interferon system. Curr Opin Microbiol. 2010;13:508–16. Epub 2010/06/12. doi: 10.1016/j.mib.2010.05.009 ; PubMed Central PMCID: PMC2920345. 20538505 10.1016/j.mib.2010.05.009 PMC2920345

[22] Kindler E, Thiel V, Weber F. Interaction of SARS and MERS coronaviruses with the antiviral interferon response. Adv Virus Res. 2016;96:219–43. doi: 10.1016/bs.aivir.2016.08.006 . 27712625 10.1016/bs.aivir.2016.08.006 PMC7112302

[23] Mielech AM, Kilianski A, Baez-Santos YM, Mesecar AD, Baker SC. MERS-CoV papain-like protease has deISGylating and deubiquitinating activities. Virology. 2014;450–451:64–70. doi: 10.1016/j.virol.2013.11.040 ; PubMed Central PMCID: PMCPMC3957432. 24503068 10.1016/j.virol.2013.11.040 PMC3957432

[24] van Boheemen S, de Graaf M, Lauber C, Bestebroer TM, Raj VS, Zaki AM, et al Genomic characterization of a newly discovered coronavirus associated with acute respiratory distress syndrome in humans. MBio. 2012;3:e00473–12. Epub 2012/11/22. doi: 10.1128/mBio.00473-12 ; PubMed Central PMCID: PMC3509437. 23170002 10.1128/mBio.00473-12 PMC3509437

[25] Corman VM, Kallies R, Philipps H, Gopner G, Muller MA, Eckerle I, et al Characterization of a novel betacoronavirus related to middle East respiratory syndrome coronavirus in European hedgehogs. J Virol. 2014;88:717–24. doi: 10.1128/JVI.01600-13 ; PubMed Central PMCID: PMCPMC3911734. 24131722 10.1128/JVI.01600-13 PMC3911734

[26] Menachery VD, Mitchell HD, Cockrell AS, Gralinski LE, Yount BL Jr., Graham RL, et al MERS-CoV Accessory ORFs play key role for infection and pathogenesis. MBio. 2017;8:e00665–17. doi: 10.1128/mBio.00665-17 ; PubMed Central PMCID: PMCPMC5565963. 28830941 10.1128/mBio.00665-17 PMC5565963

[27] Totura AL, Baric RS. SARS coronavirus pathogenesis: host innate immune responses and viral antagonism of interferon. Curr Opin Virol. 2012;2:264–75. Epub 2012/05/11. doi: 10.1016/j.coviro.2012.04.004 . 22572391 10.1016/j.coviro.2012.04.004 PMC7102726

[28] Thornbrough JM, Jha BK, Yount B, Goldstein SA, Li Y, Elliott R, et al Middle East respiratory syndrome coronavirus NS4b protein inhibits host RNase L activation. MBio. 2016;7:e00258 doi: 10.1128/mBio.00258-16 ; PubMed Central PMCID: PMCPMC4817253. 27025250 10.1128/mBio.00258-16 PMC4817253

[29] Sola I, Almazán F, Zúñiga S, Enjuanes L. Continuous and discontinuous RNA synthesis in coronaviruses. Annu Rev Virol. 2015;2:265–88. doi: 10.1146/annurev-virology-100114-055218 26958916 10.1146/annurev-virology-100114-055218 PMC6025776

[30] Freundt EC, Yu L, Park E, Lenardo MJ, Xu XN. Molecular determinants for subcellular localization of the severe acute respiratory syndrome coronavirus open reading frame 3b protein. J Virol. 2009;83:6631–40. Epub 2009/05/01. doi: 10.1128/JVI.00367-09 ; PubMed Central PMCID: PMC2698541. 19403678 10.1128/JVI.00367-09 PMC2698541

[31] McBride R, van Zyl M, Fielding BC. The coronavirus nucleocapsid is a multifunctional protein. Viruses. 2014;6:2991–3018. doi: 10.3390/v6082991 ; PubMed Central PMCID: PMCPMC4147684. 25105276 10.3390/v6082991 PMC4147684

[32] Lokugamage KG, Narayanan K, Nakagawa K, Terasaki K, Ramirez SI, Tseng CT, et al Middle East Respiratory Syndrome coronavirus nsp1 inhibits host gene expression by selectively targeting mRNAs transcribed in the nucleus while sparing mRNAs of cytoplasmic origin. J Virol. 2015;89:10970–81. Epub 2015/08/28. doi: 10.1128/JVI.01352-15 ; PubMed Central PMCID: PMCPMC4621111. 26311885 10.1128/JVI.01352-15 PMC4621111

[33] Zhang Q, Shi K, Yoo D. Suppression of type I interferon production by porcine epidemic diarrhea virus and degradation of CREB-binding protein by nsp1. Virology. 2016;489:252–68. doi: 10.1016/j.virol.2015.12.010 . 26773386 10.1016/j.virol.2015.12.010 PMC7111358

[34] Hiscox JA. The interaction of animal cytoplasmic RNA viruses with the nucleus to facilitate replication. Virus Res. 2003;95:13–22. . 12921992 10.1016/S0168-1702(03)00160-6 PMC7125749

[35] Le Sage V, Mouland AJ. Viral subversion of the nuclear pore complex. Viruses. 2013;5:2019–42. doi: 10.3390/v5082019 ; PubMed Central PMCID: PMCPMC3761240. 23959328 10.3390/v5082019 PMC3761240

[36] Malik P, Tabarraei A, Kehlenbach RH, Korfali N, Iwasawa R, Graham SV, et al Herpes simplex virus ICP27 protein directly interacts with the nuclear pore complex through Nup62, inhibiting host nucleocytoplasmic transport pathways. J Biol Chem. 2012;287:12277–92. doi: 10.1074/jbc.M111.331777 ; PubMed Central PMCID: PMCPMC3320978. 22334672 10.1074/jbc.M111.331777 PMC3320978

[37] Chang CW, Lee CP, Su MT, Tsai CH, Chen MR. BGLF4 kinase modulates the structure and transport preference of the nuclear pore complex to facilitate nuclear import of Epstein-Barr virus lytic proteins. J Virol. 2015;89:1703–18. doi: 10.1128/JVI.02880-14 ; PubMed Central PMCID: PMCPMC4300756. 25410863 10.1128/JVI.02880-14 PMC4300756

[38] Frieman M, Yount B, Heise M, Kopecky-Bromberg SA, Palese P, Baric RS. Severe acute respiratory syndrome coronavirus ORF6 antagonizes STAT1 function by sequestering nuclear import factors on the rough endoplasmic reticulum/Golgi membrane. J Virol. 2007;81:9812–24. doi: 10.1128/JVI.01012-07 . 17596301 10.1128/JVI.01012-07 PMC2045396

[39] Zhao J, Falcon A, Zhou H, Netland J, Enjuanes L, Perez Brena P, et al Severe acute respiratory syndrome coronavirus protein 6 is required for optimal replication. J Virol. 2009;83:2368–73. doi: 10.1128/JVI.02371-08 . 19091867 10.1128/JVI.02371-08 PMC2643704

[40] Zhou H, Ferraro D, Zhao J, Hussain S, Shao J, Trujillo J, et al The N Terminal Region of Severe Acute Respiratory Syndrome Coronavirus Protein 6 Induces Membrane Rearrangement and Enhances Virus Replication. J Virol. 2010. Epub 2010/01/29. doi: JVI.02570-09 [pii] doi: 10.1128/JVI.02570-09 . 20106914 10.1128/JVI.02570-09 PMC2838104

[41] Reid SP, Leung LW, Hartman AL, Martinez O, Shaw ML, Carbonnelle C, et al Ebola virus VP24 binds karyopherin alpha1 and blocks STAT1 nuclear accumulation. J Virol. 2006;80:5156–67. doi: 10.1128/JVI.02349-05 ; PubMed Central PMCID: PMCPMC1472181. 16698996 10.1128/JVI.02349-05 PMC1472181

[42] Wang C, Sun M, Yuan X, Ji L, Jin Y, Cardona CJ, et al Enterovirus 71 suppresses interferon responses by blocking Janus kinase (JAK)/signal transducer and activator of transcription (STAT) signaling through inducing karyopherin-alpha1 degradation. J Biol Chem. 2017;292:10262–74. doi: 10.1074/jbc.M116.745729 ; PubMed Central PMCID: PMCPMC5473229. 28455446 10.1074/jbc.M116.745729 PMC5473229

[43] Gustin KE, Sarnow P. Effects of poliovirus infection on nucleo-cytoplasmic trafficking and nuclear pore complex composition. EMBO J. 2001;20:240–9. doi: 10.1093/emboj/20.1.240 ; PubMed Central PMCID: PMCPMC140206. 11226174 10.1093/emboj/20.1.240 PMC140206

[44] Gustin KE, Sarnow P. Inhibition of nuclear import and alteration of nuclear pore complex composition by rhinovirus. J Virol. 2002;76:8787–96. doi: 10.1128/JVI.76.17.8787-8796.2002 ; PubMed Central PMCID: PMCPMC136411. 12163599 10.1128/JVI.76.17.8787-8796.2002 PMC136411

[45] Taylor SL, Frias-Staheli N, Garcia-Sastre A, Schmaljohn CS. Hantaan virus nucleocapsid protein binds to importin alpha proteins and inhibits tumor necrosis factor alpha-induced activation of nuclear factor kappa B. J Virol. 2009;83:1271–9. doi: 10.1128/JVI.00986-08 ; PubMed Central PMCID: PMCPMC2620888. 19019947 10.1128/JVI.00986-08 PMC2620888

[46] Ye J, Chen Z, Li Y, Zhao Z, He W, Zohaib A, et al Japanese encephalitis virus NS5 inhibits type I interferon (IFN) production by blocking the nuclear translocation of IFN regulatory factor 3 and NF-κB. J Virol. 2017;91:e00039–17. doi: 10.1128/JVI.00039-17 ; PubMed Central PMCID: PMCPMC5375679. 28179530 10.1128/JVI.00039-17 PMC5375679

[47] Xu H, Su C, Pearson A, Mody CH, Zheng C. Herpes simplex virus 1 UL24 abrogates the DNA sensing signal pathway by inhibiting NF-kappaB activation. J Virol. 2017;91:e00025–17. doi: 10.1128/JVI.00025-17 ; PubMed Central PMCID: PMCPMC5355614. 28100608 10.1128/JVI.00025-17 PMC5355614

[48] Poppe M, Wittig S, Jurida L, Bartkuhn M, Wilhelm J, Muller H, et al The NF-kappaB-dependent and -independent transcriptome and chromatin landscapes of human coronavirus 229E-infected cells. PLoS Pathog. 2017;13:e1006286 doi: 10.1371/journal.ppat.1006286 ; PubMed Central PMCID: PMCPMC5386326. 28355270 10.1371/journal.ppat.1006286 PMC5386326

[49] Perlman S, Netland J. Coronaviruses post-SARS: update on replication and pathogenesis. Nat Rev Microbiol. 2009;7:439–50. Epub 2009/05/12. doi: nrmicro2147 [pii] doi: 10.1038/nrmicro2147 ; PubMed Central PMCID: PMC2830095. 19430490 10.1038/nrmicro2147 PMC2830095

[50] Sims AC, Tilton SC, Menachery VD, Gralinski LE, Schafer A, Matzke MM, et al Release of severe acute respiratory syndrome coronavirus nuclear import block enhances host transcription in human lung cells. J Virol. 2013;87:3885–902. Epub 2013/02/01. doi: 10.1128/JVI.02520-12 ; PubMed Central PMCID: PMCPmc3624188. 23365422 10.1128/JVI.02520-12 PMC3624188

[51] Pewe L, Zhou H, Netland J, Tangudu C, Olivares H, Shi L, et al A severe acute respiratory syndrome-associated coronavirus-specific protein enhances virulence of an attenuated murine coronavirus. J Virol. 2005;79:11335–42. doi: 10.1128/JVI.79.17.11335-11342.2005 . 16103185 10.1128/JVI.79.17.11335-11342.2005 PMC1193615

[52] Li K, Wohlford-Lenane CL, Channappanavar R, Park JE, Earnest JT, Bair TB, et al Mouse-adapted MERS coronavirus causes lethal lung disease in human DPP4 knockin mice. Proc Natl Acad Sci USA. 2017;114:E3119–E28. doi: 10.1073/pnas.1619109114 ; PubMed Central PMCID: PMCPMC5393213. 28348219 10.1073/pnas.1619109114 PMC5393213

[53] Cockrell AS, Yount BL, Scobey T, Jensen K, Douglas M, Beall A, et al A mouse model for MERS coronavirus-induced acute respiratory distress syndrome. Nat Microbiol. 2016;2:16226 doi: 10.1038/nmicrobiol.2016.226 . 27892925 10.1038/nmicrobiol.2016.226 PMC5578707

[54] Yoshikawa T, Hill TE, Yoshikawa N, Popov VL, Galindo CL, Garner HR, et al Dynamic innate immune responses of human bronchial epithelial cells to severe acute respiratory syndrome-associated coronavirus infection. PLoS One. 2010;5:e8729 Epub 2010/01/22. doi: 10.1371/journal.pone.0008729 ; PubMed Central PMCID: PMC2806919. 20090954 10.1371/journal.pone.0008729 PMC2806919

[55] Fehr AR, Channappanavar R, Jankevicius G, Fett C, Zhao J, Athmer J, et al The conserved coronavirus macrodomain promotes virulence and suppresses the innate immune response during severe acute respiratory syndrome coronavirus infection. MBio. 2016;7:e01721–16. doi: 10.1128/mBio.01721-16 ; PubMed Central PMCID: PMCPMC5156301. 27965448 10.1128/mBio.01721-16 PMC5156301

[56] Livak KJ, Schmittgen TD. Analysis of relative gene expression data using real-time quantitative PCR and the 2(-Delta Delta C(T)) Method. Methods. 2001;25:402–8. Epub 2002/02/16. doi: 10.1006/meth.2001.1262 S1046-2023(01)91262-9 [pii]. . 11846609 10.1006/meth.2001.1262

[57] Suzuki K, Bose P, Leong-Quong RYY, Fujita DJ, Riabowol K. REAP: A two minute cell fractionation method. BMC Res Notes. 2010;3:294 doi: 10.1186/1756-0500-3-294 ; PubMed Central PMCID: PMCPMC2993727. 21067583 10.1186/1756-0500-3-294 PMC2993727

