# Supplementary figures and images for: MERS-CoV 4b protein interferes with the NF-κB-dependent innate immune response during infection
Source: PLoS Pathog. 2018 Jan 25;14(1):e1006838. doi: 10.1371/journal.ppat.1006838 (PMC5800688; doi:10.1371/journal.ppat.1006838)

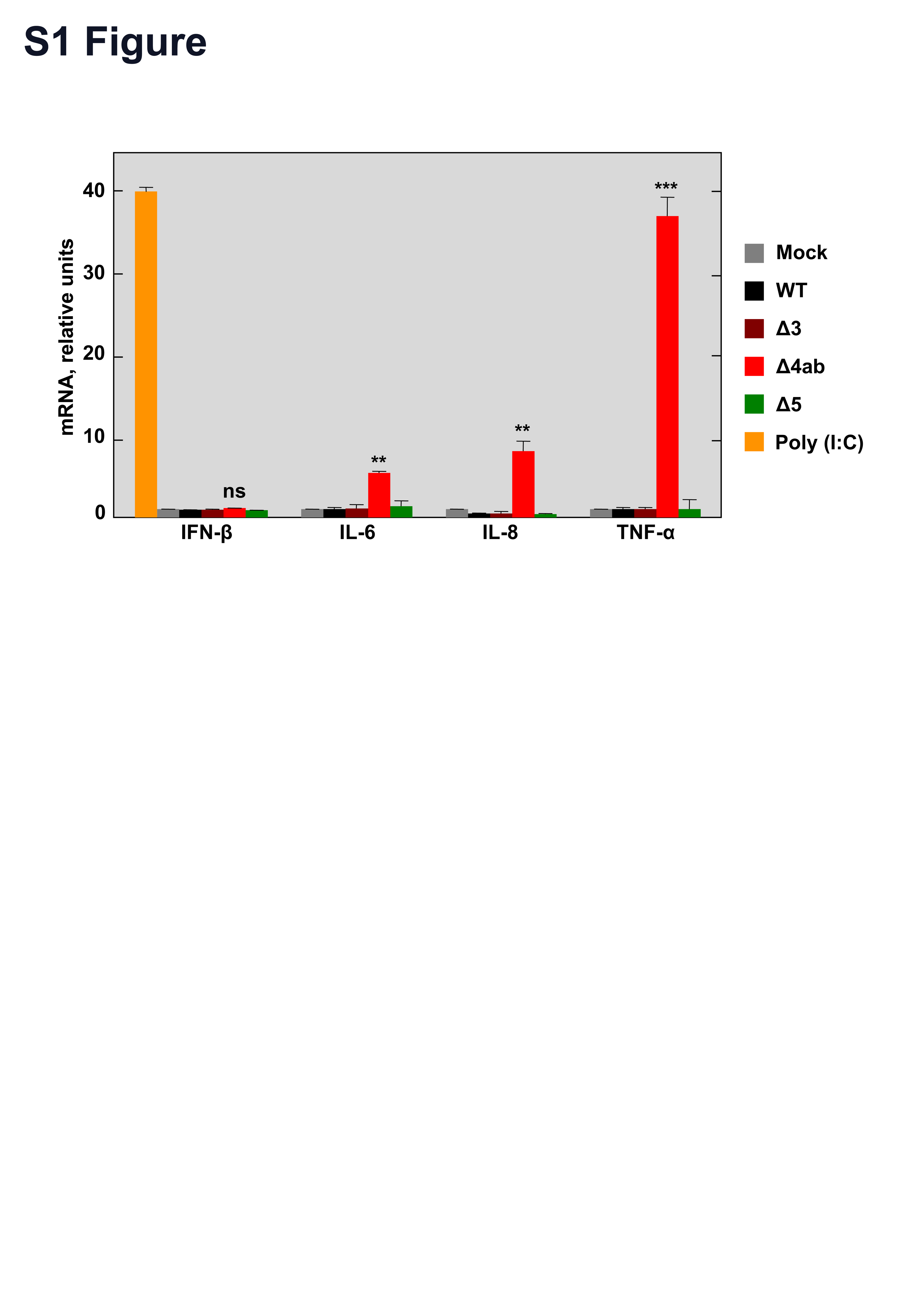

Supplement: S1 Fig — Huh-7 cells were either transfected with 2 μg poly(I:C) or infected (MOI = 1 PFU/cell) and total RNA was collected at 16 hpt or 24 hpi, respectively. IFN-β, IL-6, IL-8 and TNF-α mRNA expression levels were quantified by RT-qPCR and related to those in WT-infected cells, using the ΔΔCt method for calculation and HMBS as a reference endogenous gene. Shown are means with standard deviations, which were analyzed using an unpaired t-test against the wild-type (ns, not significant; **, p<0.01; ***, p<0.001). (TIF) [file ppat.1006838.s001.tif]

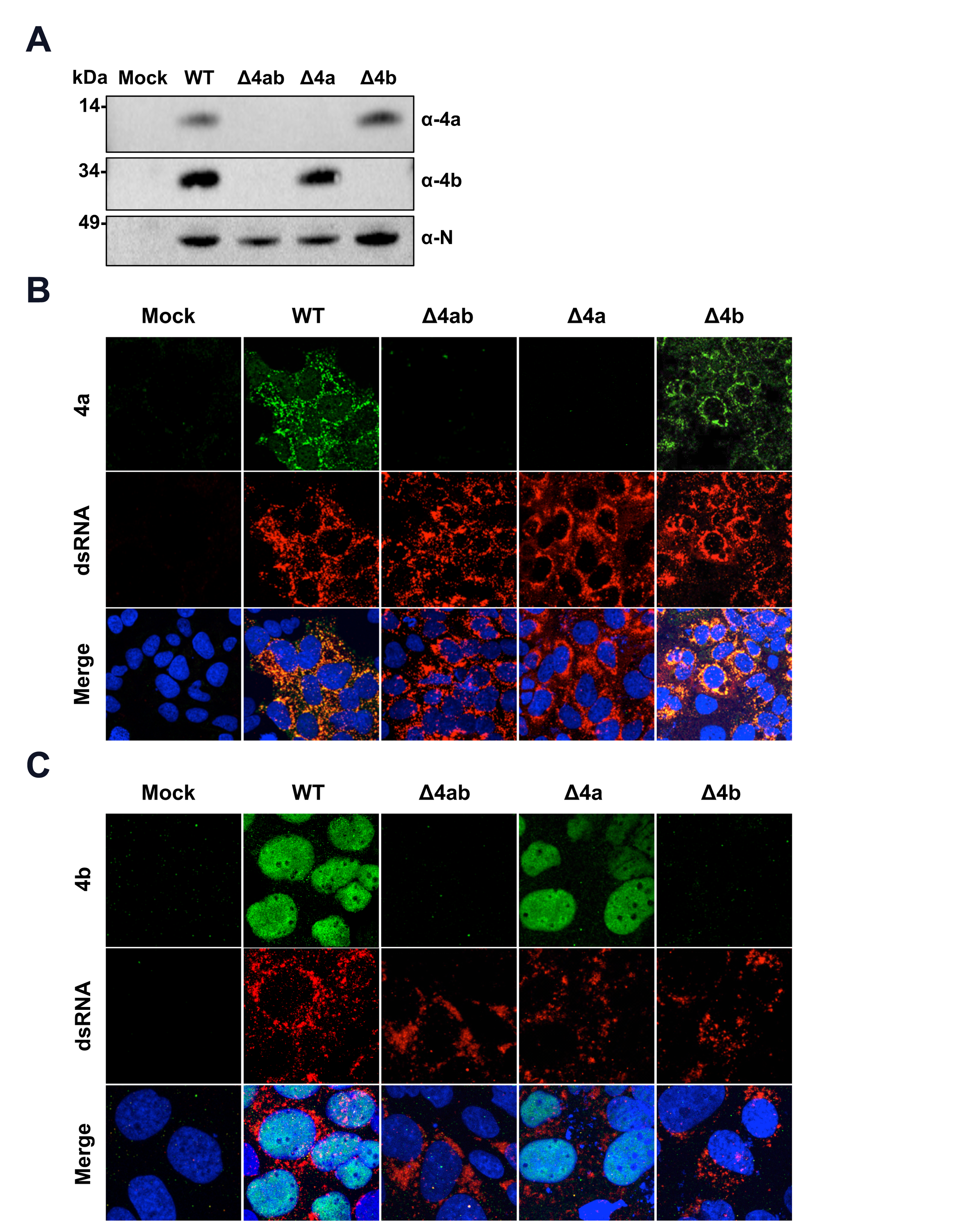

Supplement: S2 Fig — Huh-7 cells were mock-infected or infected with the WT, Δ4ab, Δ4a or Δ4b deletion mutants (MOI = 0.1 PFU/cell). At 24 hpi, cell lysates were analyzed by Western blot and detected with the indicated antibodies (A). MERS-CoV protein N was used as a positive control for infection. At 24 hpi cells were fixed and stained with specific antibodies against 4a (B) or 4b (C) (green) and dsRNA (red). Cell nuclei were stained with DAPI (blue). (TIF) [file ppat.1006838.s002.tif]

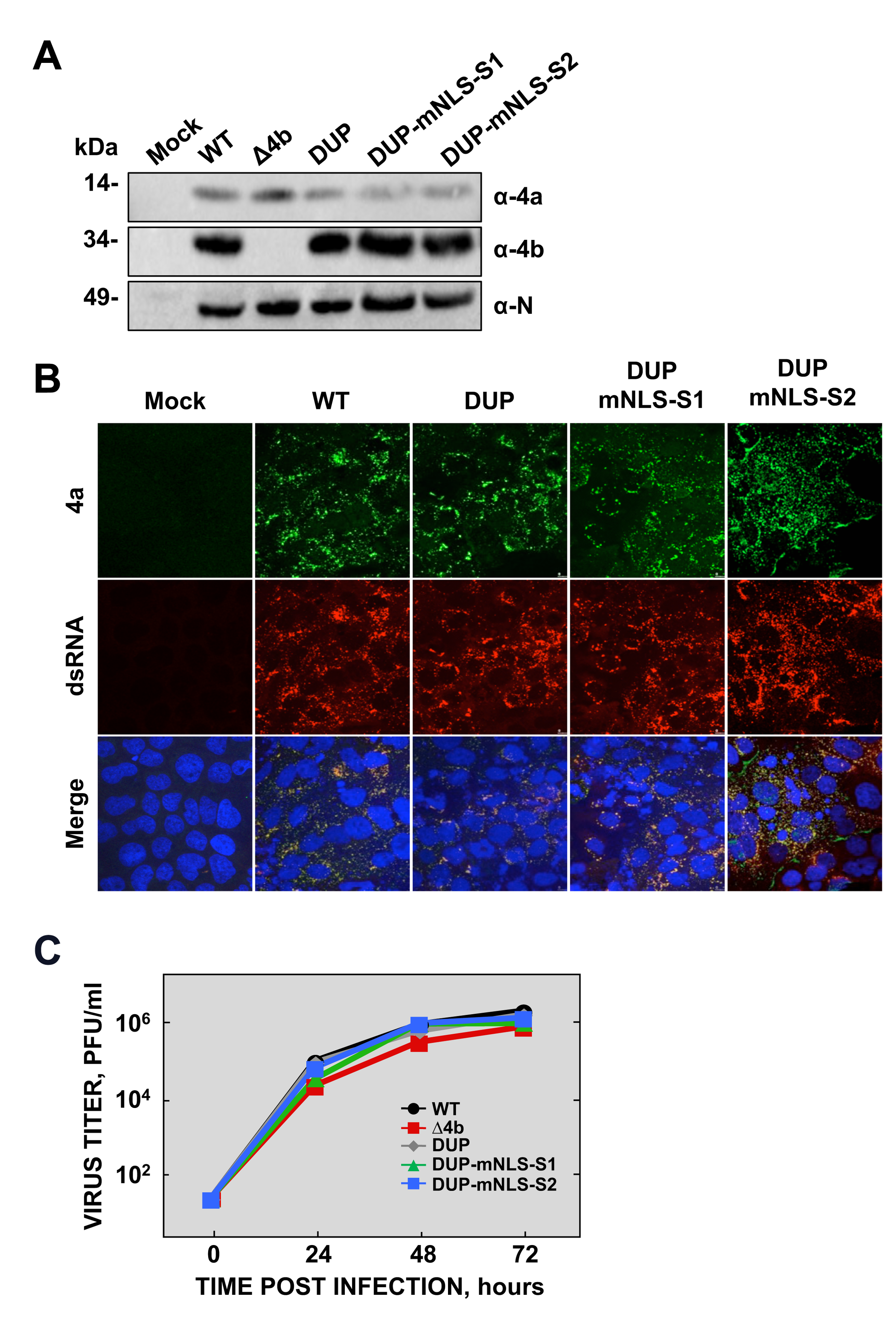

Supplement: S3 Fig — Huh-7 cells were mock-infected or infected with the WT or 4b-NLS mutants (MOI of 0.1 PFU/cell). At 24 hpi, cell lysates were analyzed by Western blot (A) with the indicated antibodies; or cells were fixed and stained with specific antibodies (B) against 4a (green) and dsRNA (red). Cell nuclei were stained with DAPI (blue). (C) Growth kinetics of MERS-CoV-4b-NLS mutants at a MOI 0.001 PFU/cell. Supernatants were collected at 24, 48 and 72 hpi and titrated by plaque assay. (TIF) [file ppat.1006838.s003.tif]

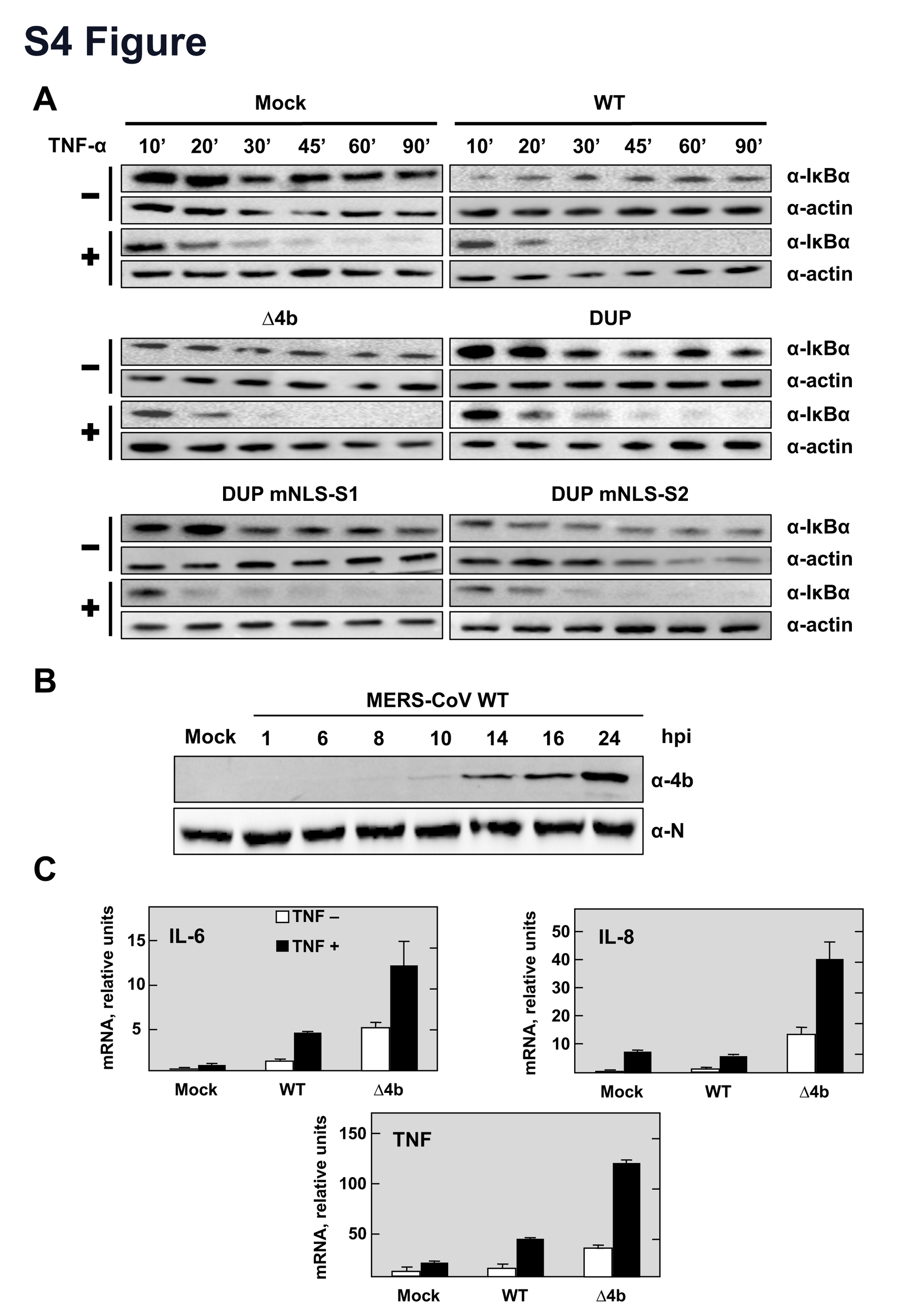

Supplement: S4 Fig — (A) Huh-7 cells were mock-infected or infected with WT, Δ4b or 4b-NLS mutants (MOI = 1 PFU/ml). At 14 hpi, cell supernatant was replaced by fresh medium containing TNF-α (50 ng/ml). After the indicated times of treatment, cell lysates were prepared for immunoblotting with anti-IκBα antibodies. Actin was used as a loading control. (B) Huh-7 cells were mock-infected or infected with WT virus (MOI = 1 PFU/cell). After indicated times, cell lysates were collected for Western blot analysis of 4b protein expression. N protein was used as a control. (C) Huh-7 cells were mock-infected or infected with WT or Δ4b mutant (MOI = 1 PFU/ml). At 14 hpi, cells were treated with TNF-α (50 ng/ml) for 30 min. Total RNA was extracted and mRNA expression levels of TNF-α, IL-6 and IL-8 were quantified by RT-qPCR and compared to those in untreated WT-infected cells, using the ΔΔCt method and HMBS as a reference endogenous gene. Error bars represent SD. (TIF) [file ppat.1006838.s004.tif]

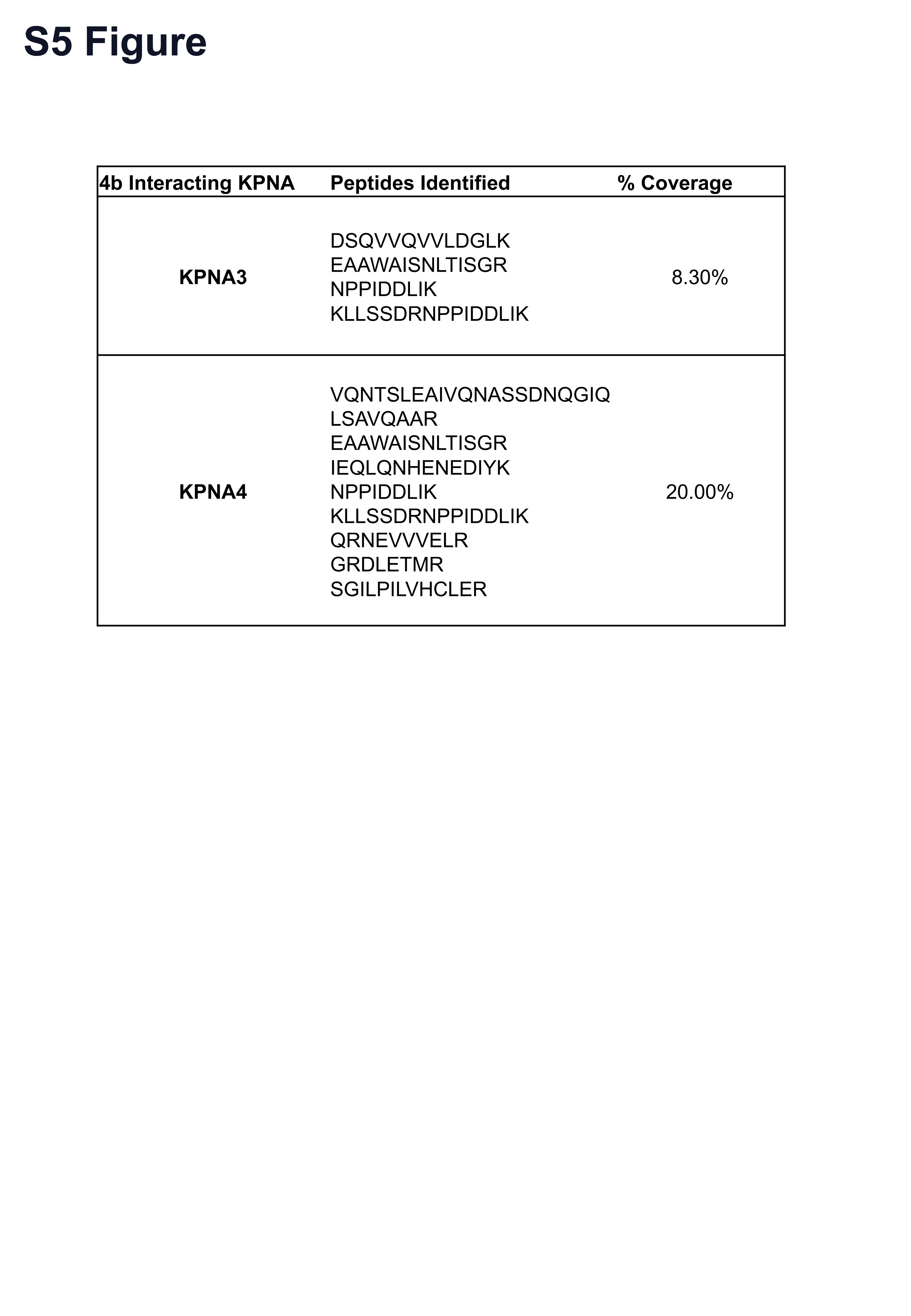

Supplement: S5 Fig — KPNA3 and KPNA4 peptides specifically identified by mass spec from 4b-FLAG Co-IP samples are listed. (TIF) [file ppat.1006838.s005.tif]

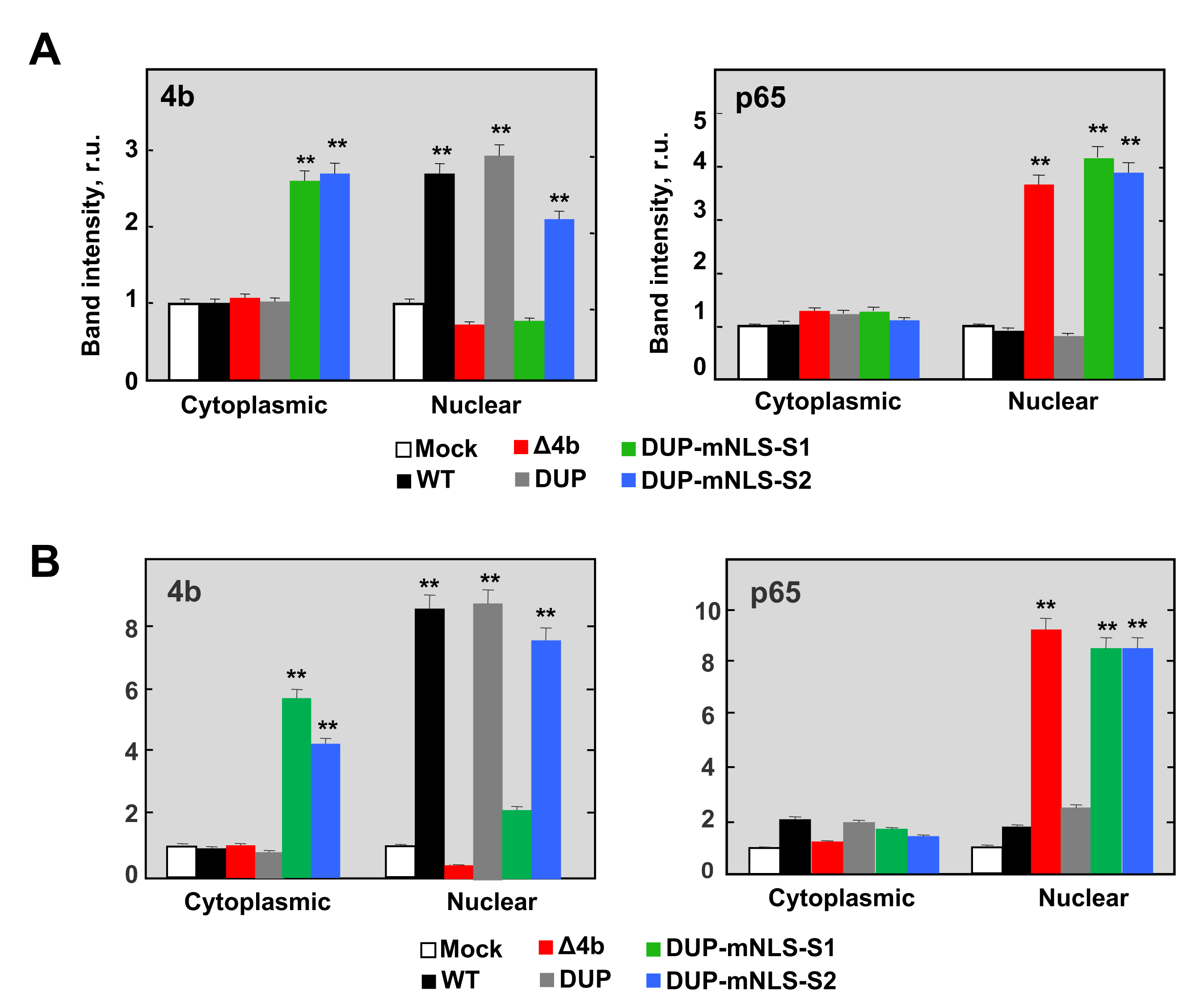

Supplement: S6 Fig — Protein 4b and NF-κB band intensities in cytoplasmic or nuclear fractions of Huh-7 (A) or Calu-3 (B) cells were normalized to GAPDH or H3 levels, respectively. The normalized intensity of 4b or p65 in the mock-infected samples was set to 1. These data represent the average results of 2 independent experiments. Shown are means with standard deviations, which were analyzed using an unpaired t-test against the wild-type (**, p<0.01). (TIF) [file ppat.1006838.s006.tif]
